# Device Architecture for Visible and Near-Infrared Photodetectors Based on Two-Dimensional SnSe_2_ and MoS_2_: A Review

**DOI:** 10.3390/mi11080750

**Published:** 2020-07-31

**Authors:** Emma P. Mukhokosi, Gollakota V.S. Manohar, Tadaaki Nagao, Saluru B. Krupanidhi, Karuna K. Nanda

**Affiliations:** 1 Materials Research Center, Indian Institute of Science, Bengaluru 560012, India; manohar93.gvs@gmail.com (G.V.S.M.); sbk@iisc.ac.in (S.B.K.); nanda@iisc.ac.in (K.K.N.); 2 Department of Physics, Muni University, P. O. Box 725 Arua, Uganda; 3 International Center for Materials Nanoarchitectonics, National Institute for Materials Science, Tsukuba 305-0044, Japan; NAGAO.Tadaaki@nims.go.jp; 4 Department of Condensed Matter Physics, Graduate School, Hokkaido University, Sapporo 060-0810, Japan

**Keywords:** device architecture, photodetectors, response speed, mobility, SnSe_2_, MoS_2_, heterostructures, graphene

## Abstract

While band gap and absorption coefficients are intrinsic properties of a material and determine its spectral range, response time is mainly controlled by the architecture of the device and electron/hole mobility. Further, 2D-layered materials such as transition metal dichalogenides (TMDCs) possess inherent and intriguing properties such as a layer-dependent band gap and are envisaged as alternative materials to replace conventional silicon (Si) and indium gallium arsenide (InGaAs) infrared photodetectors. The most researched 2D material is graphene with a response time between 50 and 100 ps and a responsivity of <10 mA/W across all wavelengths. Conventional Si photodiodes have a response time of about 50 ps with maximum responsivity of about 500 mA/W at 880 nm. Although the responsivity of TMDCs can reach beyond 10^4^ A/W, response times fall short by 3–6 orders of magnitude compared to graphene, commercial Si, and InGaAs photodiodes. Slow response times limit their application in devices requiring high frequency. Here, we highlight some of the recent developments made with visible and near-infrared photodetectors based on two dimensional SnSe_2_ and MoS_2_ materials and their performance with the main emphasis on the role played by the mobility of the constituency semiconductors to response/recovery times associated with the hetero-structures.

## 1. Introduction

**Figure 1:**
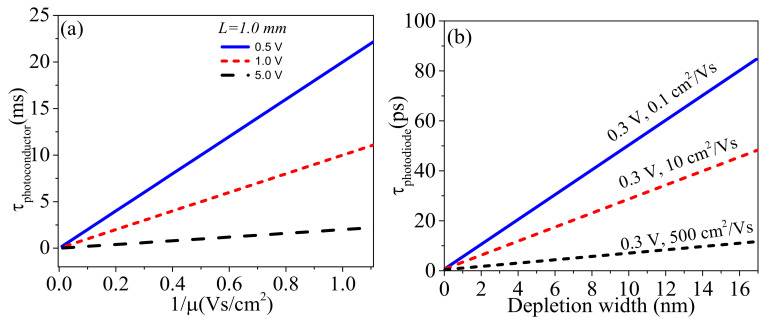
(**a**) Dependence of transit time on inverse of mobility for a photoconductor at constant electrode spacing *L* = 1.00 mm and different bias, (**b**) Dependence of transit time on depletion width for a photodiode at constant bias and different mobilities. The transit time strongly depends on mobility and bias for a photoconductor where as it strongly depends on the width of the depletion region and mobility for a photodiode. (**a**,**b**) are reproduced with permission from reference [5].

Photodetectors form vital components of many electrical and opto-electronic devices as they facilitate the conversion of light into an electric signal that can be processed by standard read-out electronics. They have found various applications such as in spectroscopy, broad-range infrared detection for night vision, fiber-optic communication, visible light detection for digital camera and video imaging and x-rays for biomedical imaging, etc. [1,2,3,4]. High response time photodetectors find applications mainly in telecommunication and have been aggressively pursued. The response time is mainly controlled by the charge carrier mobility of the semiconductor, electrode distance in the case of linear devices, and the depletion width in the case of non-linear devices, as illustrated from Figure 1a,b.

In the past, different kinds of photo-detector devices with different operating principles have been pursued. These include photoconductors [6], photodiodes [7], photoelectrochemical devices [8], phototransistors [9,10], and others which have been integrated with piezoelectric nano-generators for self-powered photodetection, which requires piezo-electric materials such as ZnO, GaN, and InN [11,12,13,14]. Conventional Si and InGaAs photodiodes have a response time of about 50 ps [6] and a responsivity of about 500 mA/W at maximum wavelength of 880 nm for Si photodiode and 1.2 A/W for InGaAs at 1550 nm [6]. However, they suffer from non-transparency, non-flexibility, limited spectral range, and relatively high manufacturing costs for InGaAs [1,15], although they can be applied for micron-scale imaging devices. Moreover, 2D layered semiconductors are a class of emerging materials with appealing properties such as transparency (at atomic level), strong light-matter interaction, good flexibility, and readiness in processing as well as in cost [6]. Graphene, the most studied 2D layered material composed of a sheet of carbon atoms just one atomic layer thick bonded together in a hexagonal honey comb lattice, was first isolated in 2004 [16]. Following the discovery, a number of its intriguing electronic, mechanical, optical and thermal properties have been studied [17]. The zero band gap and semi-metallic nature has allowed it to interact with light over a broad bandwidth from infrared (IR) to ultraviolet (UV) wavelengths and rendered it a promising material for various photodetectors over a wide spectral range. The response time of graphene can reach up to 50–100 ps [6,18], mainly due to its ballistic mobility of 2.5 × 10^5^ cm^2^/Vs [17]. However, its transparency allows it to absorb only ≈2.3% of incident visible and IR light resulting into a reported responsivity of <10 mA/W which is undesirable for high performance photodetectors and its gapless nature leads to short photo-carrier lifetime which is unfavorable for efficient photocurrent generation [19,20,21,22,23,24,25,26,27]. Although attempts have been made to improve the responsivity by introducing electron trapping centers and band-structure engineering, such as building a graphene quantum dot-like structure, this has resulted in a low response time compared to the pristine one [1,28]. Other efforts of incorporating other materials like MoS_2_/Graphene and Silicon/Graphene have similarly resulted in improved responsivity of 45.5 A/W at wavelength 642 nm and 85 mA/W at 1.55 µm respectively [29,30]. Transitional metal dichalogenides (TMDCs) are layered materials with properties that are band-gap dependent. These TMDCs have a general formulae MX_2_ (M = W, Mo, Sn, Ga, In, …etc.) and X = (Se and S) and include MoS_2_ [31,32,33,34,35,36,37,38], WS_2_ [33,39,40,41], MoSe_2_ [38,42,43], WSe_2_ [33,39,40,41,44], InSe [26,45,46,47], GaSe [48,49], and In_2_Se_3_ [50] among others, while SnSe_2_ [49] is a layered material made of earth abundant Sn metal. These materials can absorb a wide range of photon energy from 0.3 (for BP) to 2.5 eV [51]. Despite the enormous strides made by researchers in the development of layered materials for opto-electronic nano-device applications with reported responsivity of >10^4^ A/W [1,52,53,54], the response time does not compete with those of the conventional photodetectors fabricated from graphene, Si, and InGaAs photodetectors. In this review, we present the recent developments with a case study of two dimensional SnSe_2_ and MoS_2_ and their related hetero-structures as photodetectors with the main emphasis on the role of mobility, electrode spacing, and depletion width to response/recovery time. Photodetectors have been developed on both flexible and non-flexible substrates. The response time is equally affected by choice of the substrate as substrates have different carrier density and mobilities. The article is organized as follows. We first introduce the mechanisms of photodetection and define the figures of merit for a photodetector. Then devices based on SnSe_2_ and its related hetero-structures on glass, ITO, SiO_2_/Si, and Si substrates will be discussed, followed by MoS_2_ devices and their related heterostructures on Si/SiO_2_, sapphire, GaN, GaAs, and p-/n-Si substrates. Finally, we conclude by suggesting methods of designing fast response time photodetector devices taking into consideration the overall performance of the device.

## 2. Photodetector Sensing Mechanisms

Photodetection devices rely on various sensing mechanisms and the devices are named accordingly. These mechanisms include photoconductive effect, photo-gating effect, photovoltaic effect, photo-thermoelectric effect, photo-bolometric effect, photo-electrochemical effect and piezo-phototronic effect [6,55,56,57,58]. We briefly discuss these mechanisms as follows.

### 2.1. Photoconductive and Photo-Gating Effect

**Figure 2:**
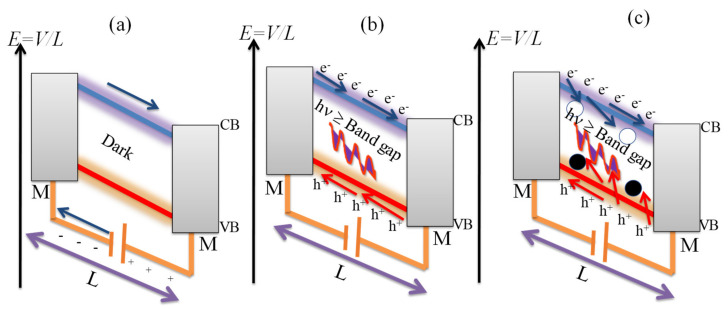
Schematic of a photoconductor between two metal contacts (M) (**a**) without illumination and (**b**) with illumination, (**c**) Schematic illustrating a photo-gating effect between a semiconductor and two Ohmic metal contacts (M) under illumination. The open and closed circles are defect states that can capture a hole or an electron eventually modulating the resistance of the semiconductor. (**a**,**b**) are adapted with permission from reference [6].

The process of photoconduction involves generation of excess free charge carriers by a semiconductor absorbing photons with energy higher than the band gap which eventually results in reduction of its electrical resistance. A photoconductor in its basic design is shown in Figure 2a and consists of a semiconductor with two Ohmic metal contacts at opposite ends.

When the device is illuminated with photons of energy greater than the band gap, as shown in Figure 2b, electron-hole pairs are generated (excitons) and are separated by an applied bias. For a linear (Ohmic) photoconductor based on a metal-semiconductor-metal structure, the transit time (considered as the charge lifetime from generation until recombination or extraction), a measure of the response time of the photodetector is defined by a mathematical relation below [5,6,59];

(1) $$\tau_{transit}=\frac{L}{\mu_{drift}\cdot E}$$

where *µ* is the mobility, *L* is the electrode spacing, and *E* is the applied field separating the free carriers. The consequence of this equation is the response time of a photoconductor highly depends on the carrier mobility of the semiconductor and electrode spacing. To achieve a short transit time requires that we use small electrode spacing and a high electric field as depicted in Figure 1a. The photoconductive gain defined as the ratio of the free photo-carrier lifetime to the transit time (*G* = τ_photocarriers_/τ_transit_) has the general expression as *G* = (τ_transit_ × *µ* × *V*)/*L*^2^. Here, one type of carrier, e.g., a hole is usually captured in a trap state with lifetime τ_photocarriers_, while the other type of carrier is free to traverse the channel with transit time of τ_transit_. Large gain results in when *τ*_photocarriers_ > *τ*_transit_ [6]. Photogating effect is an example of photoconducting effect and is due to existence of a certain amount of localized or trapped states such as defects, impurities, or surface states within the band gap of a semiconductor as illustrated in Figure 2c. These trap states can capture either the photogenerated holes or electrons and localize them and eventually act as local gates that modulate the resistance of the semiconductor [52]. This effect is common in low dimensional systems, such as TMDCs and colloidal quantum dots, which possess a large surface-to-volume ratio and reduced screening effect [4,52].

### 2.2. Photo-Electrochemical Effect

**Figure 3:**
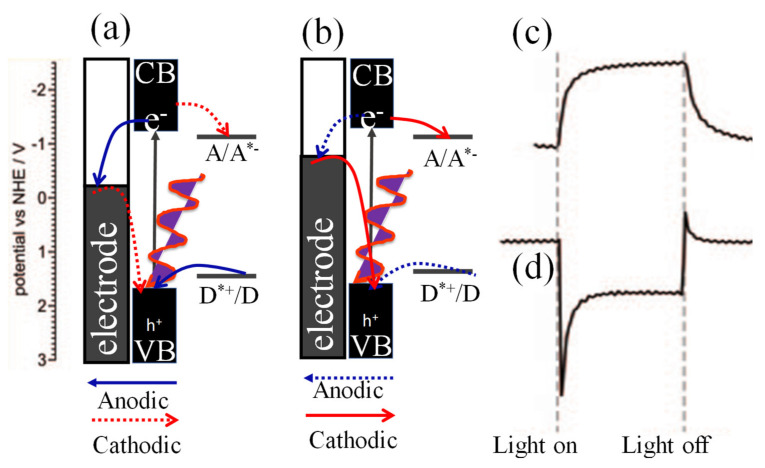
Mechanisms of anodic (**a**) and cathodic (**b**) photocurrent generation at the electrode covered with an n-semiconductor. Kinetics of anodic (**c**) and cathodic (**d**) photocurrent evolution. A/A*^−^ and D*^+^/D are acceptor and donor levels. (**a**–**c**) are adapted with permission from reference [60].

The mechanism of photocurrent switching in a photo-electrochemical device is illustrated in Figure 3a,b and depends on various parameters which involve the redox properties of the semiconductor, availability of donors and acceptors in the electrolyte, applied potentials and energy of incident photons [60]. Upon photoexcitation of the semiconductor with energy greater than the band gap, a photocurrent is generated. By considering an electrode covered with the n-type semiconductor, the photo-generated electrons from valence band to conduction band can be transferred to the electrode if its potential is higher than the potential of trapped electrons. In the presence of electron donors and acceptors such as H^+^ and (OH)^−^, interfacial electron transfer between the semiconductor and the electrolyte solution occurs. Anodic photocurrents require that an electron donor is easily oxidized by photo-generated holes and that the electrode potential enables electron transfer from the conduction band of the semiconductor to the electrode as illustrated in Figure 3a. Cathodic photocurrents occur when reduction of the electron acceptor by electrons from the conduction band and holes is more efficient than the mechanisms responsible for anodic photocurrent generation as illustrated in Figure 3b. The evolution of anodic photocurrents follows the kinetics presented in Figure 3c. The kinetics of cathodic photocurrent evolution is presented in Figure 3d [60,61,62,63].

### 2.3. Photovoltaic Effect

This can be categorized into two:

#### 2.3.1. Metal-Semiconductor Configuration (Schottky/Rectifying Metal Contacts)

**Figure 4:**
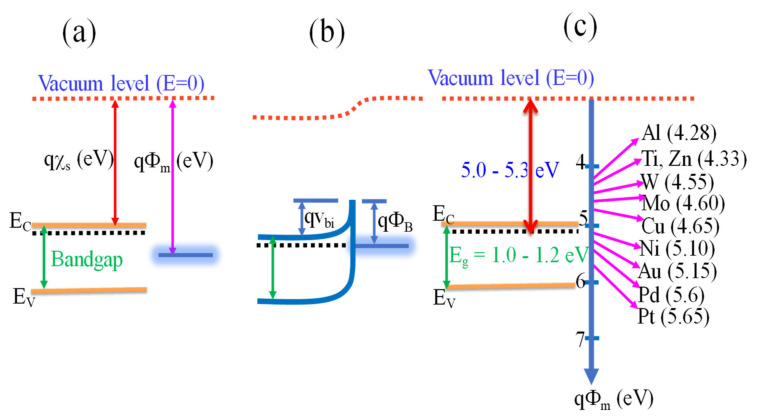
(**a**,**b**) Energy band diagram of isolated metal adjacent to isolated n-type semiconductor before equilibrium and metal-semiconductor in thermal equilibrium and (**c**) is possible metals with which SnSe_2_ can form Schottky and Ohmic metal contacts.

Here, a Schottky junction is a junction formed between a semiconductor and one of the metal electrodes [55] with a large barrier height and low doping concentration less than the density of states in the conduction or valence band [59]. The condition for the formation of a rectifying contact is based on the work function of the metal (***ϕ***_m_) and semiconductor (***ϕ***_S_) and depends on whether the semiconductor is n-type or p-type. The work function is defined as the energy difference between the Fermi and vacuum levels [59]. The potential barrier between the metal and the semiconductor is illustrated on the energy band diagram of Figure 4a,b and is the difference between the metal work function ***ϕ***_m_ and the semiconductor electron affinity ***χ***, and is given by

*q*·***ϕ***_B_ = *q* (***ϕ***_m_ − ***χ***). (2)

The electron affinity is defined as the energy difference between the conduction band edge and the vacuum level in the semiconductor [59]. For the case of an ideal contact between a metal and a p-type semiconductor, the barrier height (***ϕ***_B_) is given by

*q·**ϕ***_B_ = *qE_g_* − *q* (***ϕ***_m_ − ***χ***) (3)

where *E_g_* is the band gap of the semiconductor. For n-type semiconductor to form a Schottky diode, ***ϕ***_m_ > ***χ***. Similarly, ***ϕ***_m_ < ***χ*** for p-type semiconductor [57]. As an example, Figure 4c illustrates the possible metals with which bulk n-SnSe_2_ forms Ohmic and Schottky diode. The work function of bulk SnSe_2_ is between 5.0 and 5.3 eV [64,65].

#### 2.3.2. Semiconductor-Semiconductor Configuration (p-n, n+-n++, or p+-p++ Junctions)

**Figure 5:**
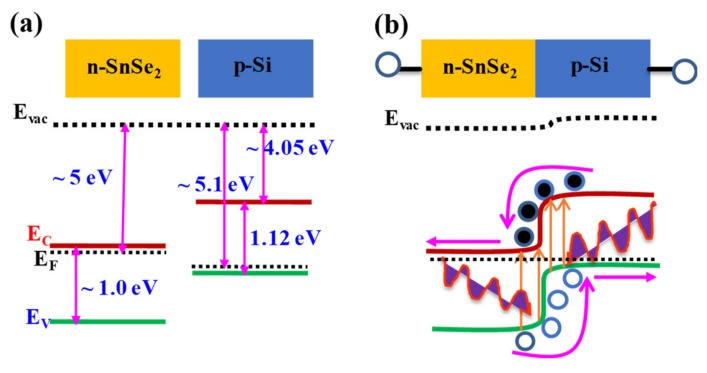
(**a**,**b**) A Energy band diagram of n-SnSe_2_ and p-Si before at equilibrium. At equilibrium, the Fermi levels of n- and p-side line up as shown by the dashed lines. The absorption of a photon with energy hν ≥ band gap will generate electron-hole pairs. The electron-hole pairs are then separated and accelerated by the built-in electric field at the junction. (**a**,**b**) are reproduced with permission from reference [66].

This effect is based on either a p–n, n–n^+^, or p–p^+^ junctions formed between either a p- and p^+^-type, n- and n^+^-type or a p- and n-type semiconductor. Here, p refers to a doped semiconductor where the majority of charge carriers are holes, n is where a majority of charge carriers are electrons while n^+^ and p^+^ have relatively larger carrier concentrations compared to n and p doped semiconductors. As an example, the formation of a p–n junction is illustrated in Figure 5a,b and is based on p-Si and n-SnSe_2_. When the device is illuminated with photons of energy greater than the band gap, electron/hole pairs are generated. The junction leads to charge carrier separation after the excitation process and electrons and holes drift in opposite directions towards the electrodes driven by the built-in electric field at the interface. The built-in electric field is normally produced at the depleted semiconductor region (Junction) where there is a significant difference in the work functions between the two materials. A photodiode displays rectifying current-voltage characteristics in the dark. The photodiode can function at two modes under illumination; i.e., photovoltaic (zero bias) and photoconductive mode (reverse bias). In photovoltaic mode, the photo-generated electron-hole pairs are separated by the built-in electric field and collected at opposite electrodes, which generates a short-circuit current (*I*_SC_). The electrical output can be open-circuit voltage (*V*_OC_). A photodiode working in photovoltaic mode has the lowest dark current leading to an improved detectivity and sensitivity. The magnitude of the reverse current increases when the device is illuminated. This is because photo-excited carriers are swept in opposite directions by the built-in electric field. The photovoltaic mode can also be used to convert the energy of the photons to electrical power (solar cell). In photoconductive mode, the external electric field is in the same direction as the built-in electric field which increases the separation efficiency of the electron-hole pairs and the response time. The charge carrier transit time for a p–n or Schottky junction depends on the width of the depletion region as well as charge carrier mobility, as depicted in Figure 1b, and is defined as [5,59]

(4) $$\tau_{transit}=\frac{W}{v_{drift}}=\frac{W}{\left[\mu_{drift}E_{o}\right]},$$

where

(5) $$W=\sqrt{\frac{\varepsilon \left(V_{bi}-V_{a}\right)\left[N_{a}+N_{d}\right]}{2\pi {eN}_{a}N_{d}}}$$

is the width of the depletion region, *V_bi_, V_a_, N_a_, N_d_, μ_drift_,* and *E_o_* are built-in potential, applied potential, concentration of acceptor atoms, concentration of donor atoms, electron-hole drift mobility and built-in electric field, respectively. The consequence of Equation (4) is that the transit time highly depends on the depletion width which is in the order of few nm and electron/hole mobility and as a result, the transit time is much faster for p–n or Schottky junction. We point out that over a certain value of the electric field, the drift velocity saturates. High frequency/speed operations require the depletion region to be thin to reduce transit time but on the other hand, to increase the responsivity or quantum efficiency the depletion layer must be sufficiently thick in order to allow a large fraction of the incident light to be absorbed. Thus, there is a trade-off between the response time and responsivity/quantum efficiency of a photodetector [59].

### 2.4. Photo-Thermoelectric and Photo-Bolometric Effects

**Figure 6:**
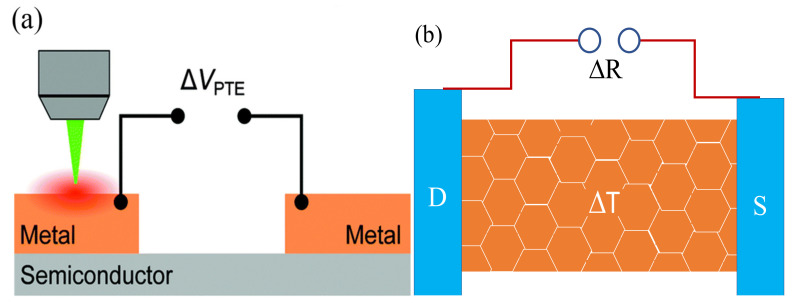
Photo-thermoelectric effect; Schematic of a semiconductor locally illuminated by a focused laser spot on one of the metal contacts to the semiconducting channel and (**b**) Schematic illustrating photo-bolometric effect. (**a**) Reproduced with permission from reference [6]. (**b**) Adapted with permission from reference [69].

Photo-thermoelectric effect refers to the generation of a temperature gradient Δ*T* from charge carriers across a semiconductor channel upon photoexcitation as illustrated in Figure 6a. The temperature gradient Δ*T* is then converted into a photo-voltage difference ΔV_PTE_ called the Seebeck effect. The magnitude of ΔV_PTE_ is determined from Δ*V*_PTE_ = Δ*T*(S_1_ − S_2_) where S_1_,_2_ are Seebeck coefficients for metal and semiconductor respectively [6,52,67]. The heat gradient mainly stems from a localized illumination with a focused laser spot. The bolometric effect refers to the change in the resistance of a material induced by heating under uniform illumination as shown in Figure 6b [4,6]. The magnitude of this effect is associated with the conductance change of photosensitive materials with temperature (dG/(dT)) and the homogeneous temperature increase (Δ*T*) caused by laser heating [52,68]. A bolometer detects the incident photon power (dP) and measures the changes in temperature (Δ*T*). The key sensing parameters are the thermal resistance *R_h_* = dT/dP and the heat capacity *C_h_*, which determines its response time, defined as *τ* = *R_h·_C_h_* [69]. The change in conductance is mainly influenced by change in carrier mobility due to the associated temperature change and or change in the number of carriers contributing to the current [69].

### 2.5. Piezo-Phototronic Effect

Piezo-phototronic is a general term that refers to devices that use piezo-potential for controlling the carrier generation, transport, separation and/or recombination for improving the performance of opto-electronic devices [55]. It requires piezo-electric materials such as ZnO, GaN, and InN, that generate an electrical potential upon variations of applied pressure/stress [11,12,13,14,55,56,57].

**Figure 7:**
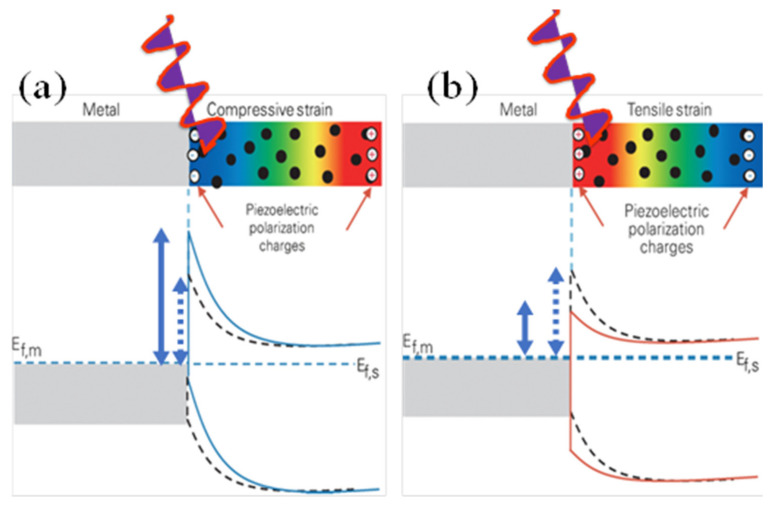
Schematic of energy diagram illustrating the effect of piezopotential on metal-semiconductor interface under illumination with photons of energy greater than the band gap; (**a**) Under compressive strain (**b**) Under tensile strain. The dotted and full double arrows indicate the changes in the SBH before and after the applied stress (piezopotential). The black dots represent the free-charge carriers in the bulk semiconductor. (**a**,**b**) are adapted with permission from reference [56].

The mechanism of operation of a piezotronic device is based on the fundamental concepts of the conventional Schottky contact and p–n junctions in semiconductor physics. The major difference is the presence of ionic charges introduced by piezoelectric polarization which can tune the carrier transport at the interface. The effect of piezopotential on metal-semiconductor contact under compressive and tensile strain is illustrated in Figure 7a,b. When a metal and an n-type piezoelectric semiconductor forms a Schottky contact (work function of metal is appreciably greater than electron affinity of the n-semiconductor) under compressive strain, as shown in Figure 7a, the negative piezoelectric polarization charges and the negative piezo potential induced at the semiconductor side can repel the electrons away from the interface, resulting in a further depleted interface and an increased local Schottky barrier height (SBH). If the piezoelectric semiconductor is under tensile strain as shown in Figure 7b, the positive piezoelectric polarization charges and the positive piezo potential created at the semiconductor side near the interface can attract the electrons toward the interface, resulting in a less depleted interface and hence a decreased local SBH. The electron-hole pairs generated through photon excitation increases conductivity and reduces Schottky barrier height due to charge redistribution [55,56,57].

## 3. Figures of Merit for Photodetectors

In this section, we define the general meaning to each figure of merit.

### 3.1. Responsivity (R)

The responsivity of a photodetector is defined as the ratio of the output photocurrent or photovoltage to the input optical power on the active region of the device. It is an indication of the achievable electrical signal under certain illumination power. A large responsivity indicates a large electrical output signal for a defined optical excitation power. It is usually expressed as *R* = *I*/(*P* × *A*), where *A* is the effective surface area, P is the power density, and *I* = *I*_illumination_ − *I*_dark_ is the photocurrent. It is measured in A/W [4,70].

### 3.2. External Quantum Efficiency (EQE)

The external quantum efficiency (*EQE*) is the ratio of the number of electron-hole pairs with contribution to the photocurrent, *n_e_* to the total number of incident photons *n*_photons_. It can be expressed as *EQE* = *n_e_*/*n*_photons_ = *R_λ_hc*/*eλ*, where *e* is the elementary charge, *h* is Planck’s constant, *c* is the speed of incident light and λ is the wavelength of incident light. The *EQE* is the measure of the optical gain G in the photodetector. *EQE* > 1 means, more than one charge carrier per impinging photon is measured. To achieve a large *EQE* in a photodetector, the optical absorption of the active layer should be high, while the carrier recombination and trapping before being collected should be minimized [4,10,23].

### 3.3. Response/Recovery Time

The response/recovery time of a photodetector is usually measured between 10% (90%) to 90% (10%) of the generated signal under modulated excitation intensity, either on the rising or falling edge. A photodetector with a small response time is usually desired for certain applications, like video-rate imaging and optical communication [6,27].

### 3.4. Noise Equivalent Power (NEP):

This is the minimum detectable optical power at which the electrical signal-to-noise ratio (SNR) in the detector is equal to unity, when bandwidth is limited to 1 Hz. NEP describes the sensitivity of a detector and is defined as the ratio of noise current to responsivity, $NEP=\frac{I_{noise}}{R}$ [1,4].

### 3.5. Detectivity (D*)

The detectivity D* is a useful parameter for comparing the detection performance of photodetectors with different materials and geometries. A higher detectivity indicates a better photodetector performance. It is defined as the reciprocal of the noise equivalent power (NEP), i.e., the minimum optical power which can be detected by the photodiodes, i.e.,

(6) $$D^{*}=\frac{\sqrt{AB}}{NEP}=\frac{R\sqrt{AB}}{S_{n}}=\frac{R\sqrt{A}}{S_{n}}$$

where A is the device area and B is its bandwidth and Sn is the noise spectral density [1,4].

Photodetector devices require both flexible and non-flexible substrates. The most available and preferred substrates in the development of photodetectors are glass, p- and n-Si, Si/SiO_2_ (thin insulating layer between 280 and 300 nm thick), Al_2_O_3_, GaN and GaAs. In the following section, we review photodetectors based on these substrates and the influence of mobility on response/recovery times of SnSe_2_, MoS_2_, and their related heterostructures.

## 4. Performance of Photodetectors Based on SnSe_2_

Band gap, absorption coefficient, mobility, and device architectures play important roles in photon absorption, responsivity, charge carrier transport, and separation. SnSe_2_ is n-type semiconductor with carrier concentration between 10^17^–10^19^ cm^−3^ [71]. The band gap varies between 0.9–2.04 eV [70,71,72,73,74], absorption coefficient of >10^4^ cm^−1^, and mobility between 0.6–85 cm^2^/Vs [3,71,72,75,76,77,78,79,80]. The highest mobility of 85 cm^2^/Vs was extracted from an exfoliated SnSe_2_ field effect transistor [80]. Most of the reported mobility is <10 cm^2^/Vs. Based on these parameters and SnSe_2_ being a layered material whose band gap depends on the number of layers coupled with elements that are earth abundant, it can effectively absorb photons of energy ≥1 eV suitable for various device applications. However, the low mobility may hinder its applications in devices that require high frequency operations. In the following section, we discuss various attempts made by different groups in the development of SnSe_2_ and its related heterostructures in photodetector applications.

**Figure 8:**
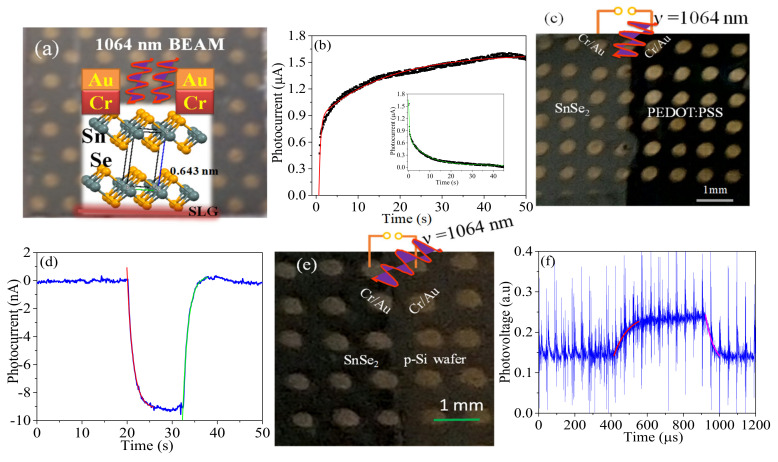
(**a**) Optical image of SnSe_2_ on soda lime glass (SLG) substrate illuminated with 1064 nm wavelength, (**b**) Response/recovery bi-exponential fitted, (**c**) Optical image of SnSe_2_/PEDOT:PSS hetero-structure on SLG substrate, (**d**) Single exponential fitted response/recovery speeds, (**e**) Optical image of SnSe_2_/p-Si hetero-structure and (**f**) Single exponential response/recovery speed of SnSe_2_/p-Si photodiode. (**a**,**b**) are reproduced with permission from reference [71], (**c**,**d**) are reproduced with permission from reference [5], (**e**,**f**) are reproduced with permission from reference [66].

Recently, our group have developed SnSe_2_ thin films on soda lime glass substrate and tuned the band gap for IR photodetection [71]. The device was illuminated with a 1064 nm wavelength, as shown in Figure 8a, and a responsivity of ~2 mA/W, and an estimated response/recovery time ~7.76 and 2.5 s at a bias of 5 and 10 V, respectively, were reported (Figure 8b) [71]. It is interesting to note that the response time is in accordance with Equation (1) for a photoconductor. We further fabricated a hetero-structure based on SnSe_2_ and PEDOT:PSS on soda lime glass substrate (Figure 8c) in order to utilize the built-in potential and narrow depletion width for fast response/recovery speed [5]. The response/recovery time improved to 1.33 and 1.22 s respectively with the device operating at zero-bias (Figure 8d) but the improve in response time resulted into a reduction in responsivity to 1.4–2.6 μA/W [5]. The fact that PEDOT:PSS and SnSe_2_ suffer from low interfacial mobility, the response/recovery time was further improved to 57 ± 25/34 ± 15 μs by fabricating a hetero-structure based on n-SnSe_2_/p-Si substrate (Figure 8e,f) and a responsivity of 120 mA/W was obtained [66]. These studies clearly demonstrate the role played by mobility/device structure in the response/recovery speeds of a photodetector. From a mobility point of view, SnSe_2_ may not compete favorably with conventional semiconductors, but its attractive layer dependent optical properties make it a viable candidate to be intergrated with conventional Si technology for various device applications.

**Figure 9:**
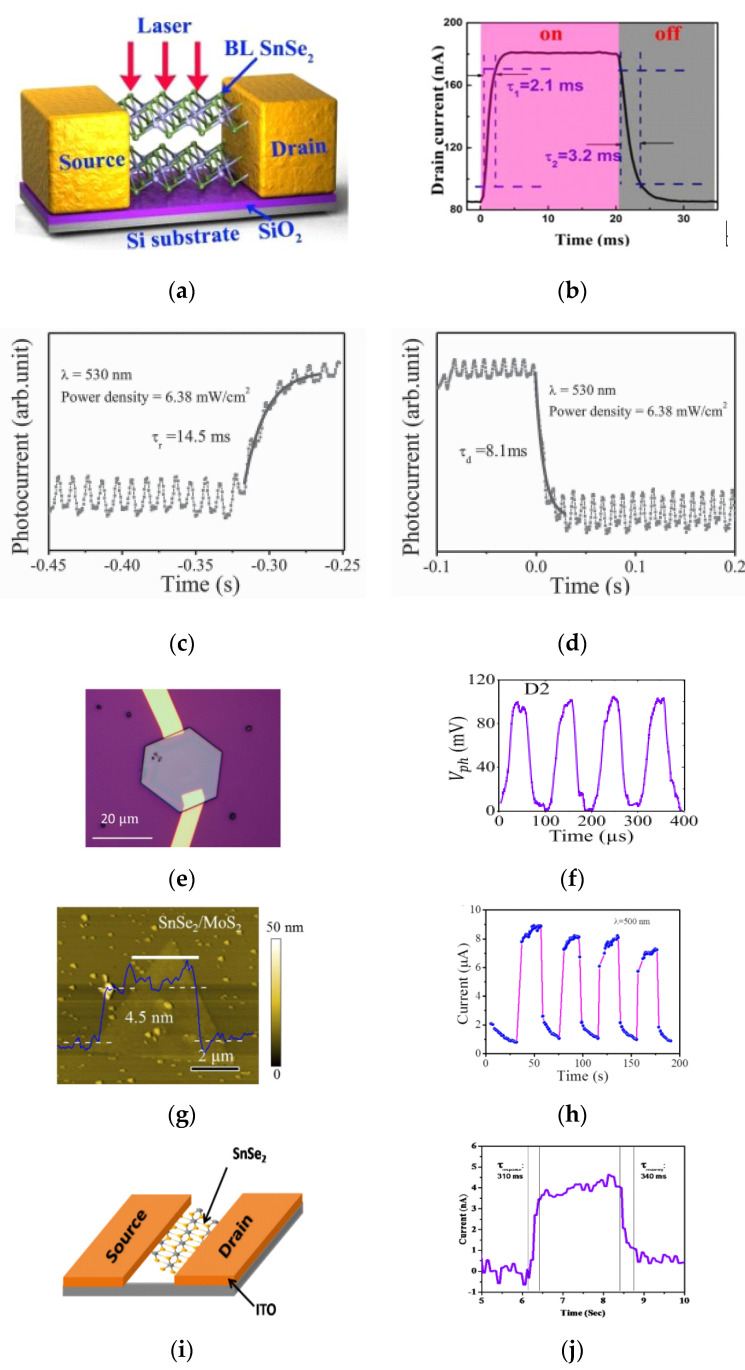
(**a**) Schematic representation of the photodetector device consisting of a bi-layered SnSe_2_ on SiO_2_/Si wafer with laser illumination. (**b**) Photocurrent dynamics of one period of the time-resolved photoresponse. The laser used in (**b**) is 633 nm with a power of 4 mW. (**a**,**b**) are reproduced with permission from reference [81]. (**c**,**d**) Rise and decay curves measured using an oscilloscope and fitted with a single-exponential function. (**c**,**d**) are reproduced with permission from reference [3]. (**e**,**f**) Optical image of electrode deposited onto a SnSe_2_ crystal (thickness ≈50 nm) to make the optoelectronic device, Rise and fall time for the SnSe_2_ photoconductor within the microsecond regime. (**e**,**f**) are reproduced with permission from reference [82]. (**g**,**h**) AFM image of a typical SnSe_2_/MoS_2_ hetero-structure, Time-dependent photoresponse of the photodetector under 500 nm light illumination. (**g**,**h**) are reproduced with permission from reference [83]. (**i**,**j**) Schematic of fabricated two probe device with few-layer SnSe_2_ and typical single *I-t* curve for response and recovery time measurements. (**i**,**j**) are reproduced with permission from reference [84].

A bilayer SnSe_2_ was mechanically exfoliated from bulk single crystals onto SiO_2_/Si substrate for photodetector and field effect transistor applications [81]. Their electrical analysis revealed a mobility of 4 cm^2^/Vs and an on/off ratio of 10^3^ at room temperature and the dark state. As shown in Figure 9a,b, the device was irradiated with a 633 nm laser beam and a fast response/recovery speed of 2.1 ± 0.3/3.2 ± 0.2 ms respectively with a responsivity of ~0.5 A/W were reported. The bilayer SnSe_2_ device had a slower response time and higher responsivity compared to that of graphene and conventional Si and InGaAs photodetectors and can mainly be attributed to low mobility of SnSe_2_. The responsivity of the bilayer device is comparable to that of conventional Si photodiodes reported at 500 mA/W at 880 nm [6], and can be attributed to high absorption coefficient of bilayer SnSe_2_. Ultrathin SnSe_2_ flakes of ~ 3 nm thick were synthesized onto mica substrate by chemical vapor deposition (CVD). The flakes were transferred onto SiO_2_/Si substrates. An indirect band gap of 1.78 eV was extracted. Electrical analysis revealed a mobility of ~0.6 cm^2^/Vs and a high on/off ratio ~2.5 × 10^3^. The device was illuminated with a 530 nm radiation and a fast response/recovery time of 14.5/8.1 ms with an ultrahigh responsivity of 1.1 × 10^3^ A/W were reported as shown in Figure 9c,d [3]. The remarkable performance of the device was attributed to the high-quality, ultrathin morphology of the SnSe_2_ flakes and the formation of the Schottky contact which increases the separation efficiency of photogenerated electron-hole pairs [3]. Using a similar CVD technique, SnSe_2_ crystal flakes of ~50 nm thick were developed on Si/SiO_2_ substrate for photodetector applications [82]. A direct band gap of ~2.0 eV and an indirect band gap of 1.0 eV were evaluated. Their electrical analysis revealed a mobility of ~0.1 cm^2^/Vs and a high-on ratio of about 100 [82]. The device was irradiated with a 543 nm radiation as shown in Figure 9e. As shown in Figure 9f, a response/recovery time of 17/45 μs with a high responsivity 0.48 A/W were reported.

It is not clear how such a high response/recovery time can be related to a device of such low mobility although the good performance of the device was mainly attributed to the formation of a Schottky barrier between SnSe_2_, SiO_2_/Si and metal contacts [82]. From mobility point of view, WSe_2_ has a higher mobility that ranges between 100–500 cm^2^/Vs [85,86,87,88,89,90,91], which is ten times more than that of SnSe_2_ (<10 cm^2^/Vs) [71]. We can assert that the higher response time of the hetero-junction is mainly attributed to the higher mobility of WSe_2_ in addition to the asymmetric band offsets at the two interfaces of the ITO/WSe_2_/SnSe_2_ hetero-junction that creates a large built-in potential across a small thickness of WSe_2_ resulting into a large built-in field [92]. A vertical photodetector device based on a SnSe_2_/MoS_2_ van der Waals hetero-structure SiO_2_/Si substrate, as shown in Figure 9g, has also been reported [83]. When the device was illuminated with a 500 nm radiation, a responsivity of 9.1 × 10^3^ A/W and response/recovery time constants of 0.2/0.6 s were obtained [83]. The transient response is shown in Figure 9h. On the basis of the mobility of MoS_2_ that has a range of values between 0.1 and 200 cm^2^/Vs depending on the method of synthesis and number of layers [93,94,95,96,97,98,99,100,101,102,103,104], one can assert that the relatively slow response time is due to low mobility of the constituent semiconductors compared to SnSe_2_/WSe_2_ hetero-structures. Compared to other findings, the authors attributed the response time to efficient charge transfer at SnSe_2_/MoS_2_ hetero-structures which were formed via epitaxial growth [83]. In a study that involves a transparent conducting oxide as a substrate, few-layer nano-sheets were developed by solvothermal approach with sheet thickness of about 3 nm [84]. A white light photodetector device was fabricated based on ITO/SnSe_2_, as shown in Figure 9i. The response/recovery time constants of the fabricated device were estimated as 310/340 ms respectively with transient response shown in Figure 9j. Manoj et al. recently have developed SnSe_2_ based photodetector on soda lime glass substrate for NIR (1064 nm) photodetection [105]. The device showed a responsivity of ~0.8 mA/W with rise and decay times of 276 ms/332 ms. The slow response times may due to the trap states present in the system. The details are summarized in Table 1.

## 5. Performance of Photodetectors Based on MoS_2_

**Figure 10:**
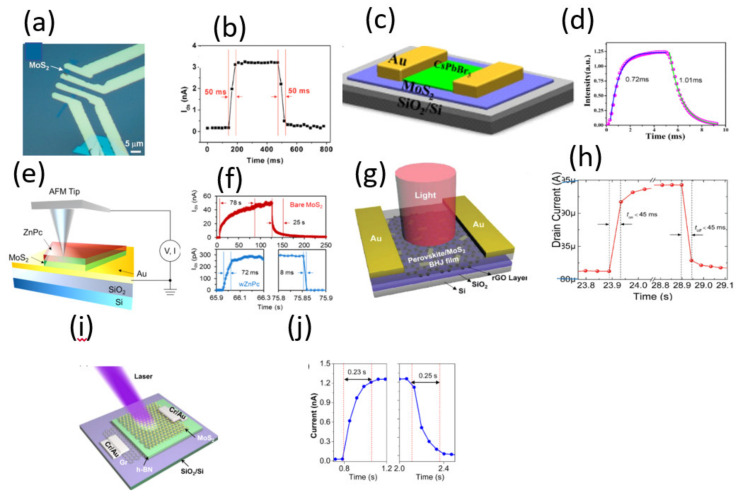
(**a**,**b**) Optical image of FET device made by single-layer MoS_2_ and photo-switching rate. Reproduced with permission from reference [107]. (**c**,**d**) Schematic illustration of the hybrid MoS_2_/CsPbBr_3_ photodetector and temporal photocurrent response of the hybrid device. Reproduced with permission from reference [108]. (**e**,**f**) Schematic of the conductive AFM measurement and time-dependent photoresponse dynamics for a MoS_2_ device after varied ZnPc treatments plotted on a linear scale. Reproduced with permission from reference [109]. Copyright 2018, American Chemical Society. (**g**,**h**) Device structure of the hybrid photodetector based on perovskite/MoS_2_ BHJ on rGO and photo-switching characteristics of the perovskite/MoS_2_ BHJ-rGO hybrid photodetector measured alternately in dark and under 660 nm light illumination (1 mW cm^−2^, VGS = 0 V, VDS = 2 V). Reproduced with permission from reference [110]. (**i**,**j**) Optical images of the device (scale bar: 10 μm), Dynamic photoresponse obtained from device. Reproduced with permission from reference [111]. QD-MoS_2_ hybrids with interfacial interaction dominated by charge transfer (left) and by non-radiative energy transfer (right), Time-resolved analysis of the normalized rise times of photocurrent at V_G_ = −40 V and V_DS_ = 0 V under 488 nm laser with 37 μW. Reproduced with permission from reference [112]. (**i**,**j**) Schematic of the MoS_2_/BN/graphene hetero-structure photodetector for photon absorber/selective hole tunneling layer/bottom electrode, respectively and Time-resolved photoresponse of the 7 h-BN nm device under 405 nm laser irradiation. Reproduced with permission from reference [113].

We next look at MoS_2_, the most studied layered transition-metal dichalcogenides with appealing optical properties that are layer dependent. A single layer is about 6.5 Å thick and has been extracted using scotch tape method by various research groups. Bulk MoS_2_ has an indirect band gap of 1.2 eV whereas single-layer MoS_2_ has a direct band gap of 1.8 eV [106]. The mobility of MoS_2_ has a range of values between 0.1 and 200 cm^2^/Vs depending on the method of synthesis and number of layers [93,94,95,96,97,98,99,100,101,102,103,104]. In this regard, a phototransistor was fabricated based on single-layer MoS_2_ by mechanical exfoliation onto Si/SiO_2_ substrate as shown in Figure 10a [107]. A carrier mobility of 0.11 cm^2^/Vs was extracted for the bottom-gate FET device configuration [107]. The device was irradiated with broad band wavelength from 450 to 800 nm. The photocurrent was higher for wavelengths lower than 670 nm which was attributed to energy greater the corresponding band gap of mono-layer MoS_2_, a photoresponsivity of ~7.8 mA/W and a response/recovery time constants 50 ms were reported with transient response shown in Figure 10b [107]. The slow response time as compared to graphene was attributed to low carrier transport [107]. In another study, a photodetector was fabricated based on monolayer MoS_2_ through mechanical exfoliation onto Si/SiO_2_ substrate [32]. The spectral response revealed a direct band gap of ~1.8 eV [32]. From their electrical analysis, a mobility of 4 cm^2^/Vs was extracted from the back-gate FET device configuration. The device was excited with a focused laser of wavelength 561 nm and a responsivity of 880 AW^−1^ at a wavelength of 561 nm was estimated. The response/recovery times were estimated as 4/9 s respectively [32]. We wish to point out that the response time reported is in accordance with the carrier mobility extracted from the device and the ultra-high responsivity can mainly be attributed to the high absorption coefficient of MoS_2_.

The response time could further be improved with devices that involve Schottky junctions and heterostructures using MoS_2_ as one of the constituent semiconductors were fabricated. Some of the fabricated devices are discussed as follows.

**Figure 11:**
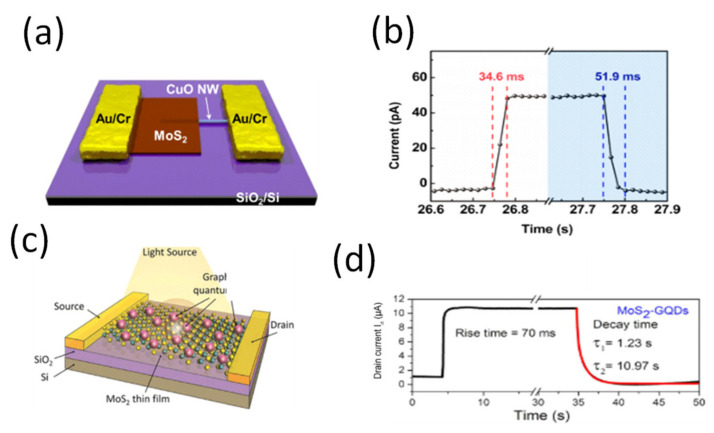
(**a**,**b**) Schematic representation of a MoS_2_/CuO nano-sheet on 1D hetero-junction photodiode, photo-responsive rise and decay times of a MoS_2_/CuO hetero-junction photodiode under light illumination of λ = 570 nm at P_light_ = 1.4 mW and a bias voltage of −2 V. Reproduced with permission from reference [138], Copyright 2016, American Chemical Society (**c**,**d**) Schematic of a MoS_2_-GQDs hetero-structure phototransistor, Time-dependent photoresponse of MoS_2_-GQDs device. Reproduced with permission from reference [142].

An ultrasensitive and broadband MoS_2_ photodetector on SiO_2_/Si substrate driven by a ferroelectric poly(vinylidene fluoride/trifluoroethylene) P(VDF-TrFE) employed to suppress the dark current of the MoS_2_ semiconducting channel has been developed [114]. The device was illuminated with a 635 nm radiation, and a responsivity of 2570 A/W with a fast response/recovery time of 1.8/2.0 ms respectively were estimated [114]. A FET mobility of 85 cm^2^/Vs was estimated from the differential *I-V* curves which is of the same order of pristine MoS_2_ [114]. The fast response time could have resulted from the strong built-in electric field at the interface of ferroelectric P(VDF-TrFE) and MoS_2_. In a study with device configuration shown in Figure 10c for MoS_2_/CsPbBr_3_ fabricated on SiO_2_/Si substrate [108], a responsivity of 4.4 A/W at 442 nm radiation with a response/recovery times of 0.72/1.01 ms were reported with transient response shown in Figure 10d. The findings were compared with the response/recovery time exclusively based on perovskite CsPbBr_3_ which was estimated 62.5/18.2 ms. By analyzing the carrier mobility of CsPbBr_3_ that ranges between ~77 and 1000 cm^2^/Vs [115,116,117,118], which is much higher than that of MoS_2_, we can assert that the difference in the rising and decay times between the perovskite photodetector with or without MoS_2_ is due to the higher mobility of CsPbBr_3_. The authors attributed this to the carrier transfer from perovskite to MoS_2_ layer which induced trap passivation on the substrate [109]. Similarly, Huo et al. developed CsPbBr_3_/MoS_2_ heterojunction phototransistor on SiO_2_/Si substrate. The device was illuminated with 442 nm radiation, a responsivity of 13.1 A/W and the response/recovery time were estimated as 2.5/1.8 ms respectively [119]. Next we analyze a study with device configuration zinc pthalocyanine (ZnPc)/MoS_2_ developed on SiO_2_/Si substrate shown in Figure 10e for a ultrafast photoresponse [109]. A comprehensive study that involves charge transfer at the interface between MoS_2_/SiO_2_/Si and ZnPc/MoS_2_/SiO_2_/Si was carried out [109]. For MoS_2_/SiO_2_/Si device, the photoresponse was observed to persist for a minute and for 40 min ZnPc-treated MoS_2_, a fast rise and decay times of 72 and 8 ms respectively were estimated. The transient photo-responses are shown in Figure 10f. The mobility values of ZnPc is of order 10^−3^–10^−4^ cm^2^/Vs and this value may not play a major role in charge transportation [120,121,122]. The slow response in MoS_2_/SiO_2_/Si was attributed to inherent defect states in MoS_2_ and the localized trap states at the MoS_2_/SiO_2_ interface in addition to inherent low mobility. The improved response time in ZnPc-decorated MoS_2_ was regarded as a result of the suppressed slow hole trapping in the above localized states [109]. The same authors improved the responsivity of the device to 430 A/W by introducing Al_2_O_3_ passivation layer that could screen charge impurity scattering [109]. The response time of the device remained fast at ~100 ms after the passivation still 100 times faster than the bare MoS_2_ device. The slightly slower response compared to ZnPc-treated MoS_2_ was attributed to the increased number of the inherent deep trap centers in MoS_2_ and at the interface that involves the minority hole trapping when Fermi level is tuned close to the conduction band [109]. A hetero-junction was fabricated, as shown in Figure 10g, based on CH_3_NH_3_PbI_3_/MoS_2_ on SiO_2_/Si substrate combined with reduced graphene oxide (r-GO) as a hole transport layer for photodetector applications [110]. The device was illuminated with a 660 nm wavelength light and a responsivity of 1.08 × 10^4^ AW^−1^ and as shown in Figure 10h, a fast response/recovery speed of shorter than 45 ms were reported [110]. The carrier mobility of CH_3_NH_3_PbI_3_ varies between 1 and 100 cm^2^/Vs for polycrystalline and single crystals respectively [123,124,125]. The carrier mobility of r-GO varies between 1.8–83 cm^2^/Vs [126,127]. These values suggest that the mobility of CH_3_NH_3_PbI_3_, r-GO, and MoS_2_ are comparatively equal and therefore, as expected, there is no much significant change in response/recovery time constants on the basis of mobility, if any, it could be due to the presence of a narrow depletion width. Avan der Waals hetero-structure photodiode based on GaSe/MoS_2_ on SiO_2_/Si substrate have been developed [111]. When the device was illuminated with a 532 nm radiation, a responsivity of ~3 A/W and a response time of 50 ms were obtained [111]. On the basis of mobility, the mobility of GaSe has been reported and varies between 0.4–50 cm^2^/Vs [128,129,130,131,132], which is of similar order to that of MoS_2_ and in accordance with the observed response/recovery time constants. A study that applies graphene as an electrode with device structure MoS_2_/h-BN/graphene was developed on SiO_2_/Si substrate where h-BN is used as an insulating layer between graphene electrode and MoS_2_ photo-absorber as shown in Figure 10i [113]. On the illumination of the device with 405 nm of light, a responsivity of 180 AW^−1^ and a response/recovery speed of 0.23/0.25 s were reported with transient response shown in Figure 10j [113]. Yang et al. developed MoS_2_/MoSe_2_ hetero-junction on SiO_2_/Si substrate [133]. A fast response time of ~10 ms and a responsivity of ~350 A/W were reported when the device was illuminated with a pulsed light of 633 nm and was faster by 1–2 orders of magnitude as compared to isolated MoSe_2_ and MoS_2_ devices [133]. In comparison to fabricated MoSe_2_ phototransistors with a bottom-gate configuration on SiO_2_/Si substrates [134], that possess a fast response/recovery speed of 1.7/2.2 ms at 650 nm radiation with a responsivity of 1.4 × 10^5^ A/W, we can assert that the fast response in the hetero-structure is due to better mobility of MoSe_2_ as compared to that of MoS_2_. The mobility of MoSe_2_ varies between 15–118 cm^2^/Vs [38,135,136,137], and is comparatively higher than that of MoS_2_. A high-performance MoS_2_/CuO nano-sheet hetero-junction shown in Figure 11a was developed on SiO_2_/Si substrate for photodetector applications [138]. As shown in Figure 11b, a fast photoresponse/recovery speed of ~34.6/51.9 ms with a responsivity of ~ 157.6 A/W were found at 570 nm illumination [138]. The mobility of CuO reported between 0.01 and 8 cm^2^/Vs [139,140,141] is lower than that of MoS_2_ suggesting no significant effect in response/recovery time constants of the hetero-structure, if any, it could be due to a strong built-in electric field at the junction. Chen et al. developed MoS_2_ photodetectors enhanced by graphene QDs as shown in Figure 11c on SiO_2_/Si substrate [142]. With a 405 nm radiation, a responsivity of ~10^4^ A/W and a response time of 70 ms and recovery speed of 1.23 s, 10.97 s were reported [142]. The transient response is shown in Figure 11d. The response/recovery speed is low yet graphene QDs are known to have a high electron mobility [143,144]. The authors attributed the slow response to either defects or charge impurity states inside the band gap or by the presence of trap states between MoS_2_ and the underlying SiO_2_ layer, which usually occurs for MoS_2_ grown by CVD [142].

**Figure 12:**
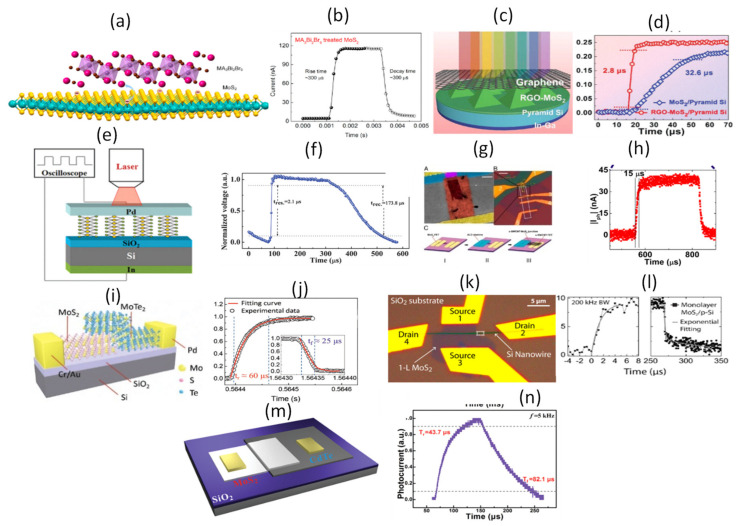
(**a**,**b**) Schematic diagram of MoS_2_ and MA_3_Bi_2_Br_9_ hetero-structure, Rise and decay time of MA_3_Bi_2_Br_9_-treated MoS_2_ device irradiated by 530 nm LED with a power density of 3.3 mW/cm^2^. Reproduced with permission from reference [145]. (**c**,**d**) Schematic illustration of the structure of 3D RGO–MoS_2_/pyramid Si hetero-junction photodetector, rise edges, Reproduced with permission from reference [146], (**e**,**f**) Schematic illustration of the setup for measuring the response time of the Pd-MoS_2_/Si device, Single normalized modulation cycle measured at 2000 Hz. Reproduced with permission from reference [147]. (**g**,**h**) False-colored SEM image of the hetero-junction diode. (Scale bar, 2.5 μm.) The yellow regions at the top and bottom are the gold electrodes. The patterned alumina (blue region) serves as a mask for insulating a portion of the SL-MoS_2_ flake (violet region). The pink region is the patterned random network of s-SWCNTs (p-type) in direct contact with the exposed part of the SL-MoS_2_ flake (n-type) to form the p-n hetero-junction diode (dark red), Time- dependent photoresponse of the p-n hetero-junction showing fast rise and decay times of ~15 μs, Reproduced with permission reference [148]. (**i**,**j**) Schematic diagram of the MoTe_2_/MoS_2_ van der Waals hetero-structure, Time resolved photoresponse of the hetero-structure at V_sd_ ≤ 0.51 V. The inset is falling edge of the response, Reproduced with permission from reference [152]. (**k**,**l**) Optical micrograph of a device in which a MoS_2_ monolayer was transferred onto a p-Si nanowire followed by contact fabrication, Transient photocurrent of monolayer MoS_2_/Si nanowire p-n hetero-junction (black squares) measured with a high-speed current preamplifier (200 kHz bandwidth) at VD = −8 V. Rise and fall times are fit with single and bi-exponentials (gray lines), and equal t_rise_ = 1.4 μs and t_fall_ = 1.6 μs (80 μs), respectively. Reproduced with permission from reference [159]. (**m**,**n**) Schematic illustration of a MoS_2_/CdTe hetero-junction device, Rising and falling edges for estimating the rise time and the fall time at 5 kHz. Reproduced with permission from reference [165].

A low noise and fast photoresponse of few-layered MoS_2_ passivated by MA_3_Bi_2_Br_9_ on SiO_2_/Si substrate has been developed as shown in Figure 12a. With a 530 nm light, a responsivity of 112 A/W and the response/recovery speed of 0.3 ms were obtained. The transient response is shown in Figure 12b [145]. Our literature search could not reveal the mobility of MA_3_Bi_2_Br_9_ but the response time suggests that it is of the same order to that of MoS_2_. The MA_3_Bi_2_Br_9_ passivation was responsible for fast and strong photo-response of the MoS_2_ [145]. A 3D rGO-MoS_2_/pyramid Si hetero-junction with device structure as shown in Figure 12c was fabricated for ultrahigh detectivity and ultra-broadband photodetection on Si/SiO_2_ substrate [146]. The device was illuminated with a 808 nm radiation and a responsivity of 21.8 A/W and a response/recovery of 2.8/46.6 μs were found [146]. The transient response is shown in Figure 12d. To elucidate on the fast response time, two devices were compared one with RGO–MoS_2_/pyramid Si and the other with MoS_2_/pyramid Si device (rise time/decay time = 32.6/87.8 µs). We wish to mention here that the carrier mobility of r-GO varies between 1.8 and 83 cm^2^/Vs [126,127]. The authors attribute the faster response time to the insertion of RGO in the device that improved conductivity of MoS_2_ film [146]. A self-powered broadband, high-detectivity and ultrafast photodetectors based on Pd-MoS_2_/Si hetero-junction with device structure shown in Figure 12e was developed [147]. The device was illuminated with a broad band radiation (300–1100 nm), a responsivity of ~654.0 mA/W at 950 nm and a response/recovery speed of 2.1/173.8 μs were reported [147]. The transient response is shown in Figure 12f. The mobility of n-Si is about 1500 cm^2^/Vs which is several orders of magnitude higher than that of MoS_2_ [59]. We believe, it is this high electron mobility and a strong built-in electric field at the interface that are response for the fast response time. A gate-tunable carbon nanotube–MoS_2_ hetero-junction p–n diode has been developed on SiO_2_/Si substrate as shown in Figure 12g [148]. The device was illuminated with a 650 nm radiation, a responsivity exceeding 0.1 A/W with a response time of 15 μs (limited by the instrument set-up) was reported [148]. The transient response is shown in Figure 12h. On the basis of mobility, carbon nano tubes (CNTs) are reported to have a high mobility of >10^4^ cm^2^/Vs [149,150], several orders of magnitude higher than that of MoS_2_. We believe it is this extraordinary high mobility of CNTs that is primarily response for the fast response of the device. A high-performance photovoltaic detector based on MoTe_2_/MoS_2_ van der Waals hetero-structure shown in Figure 12i was fabricated on SiO_2_/Si substrate [151]. The device was illuminated with a 637 nm radiation, a responsivity of 46 mA/W and a response/recovery speed of 60/25 μs was reported [151]. The transient response is shown in Figure 12j. The carrier mobility of MoTe_2_ is of ranges between 0.3–4000 cm^2^/Vs [152,153,154,155,156], depending on the preparation conditions. This value suggests that the mobility of MoTe_2_ is greater than that of MoS_2_ and this explains the higher response time observed in the heterostructure in addition to the strong built-in electric field. Henning et al. fabricated a mixed-dimensional single and multilayer MoS_2_/p-silicon nanowire hetero-junction with device structure shown in Figure 12k on SiO_2_/Si substrate and studied the charge separation at the junction. [157] They carried out time-resolved photocurrent measurements on four types of devices: p-Si nanowire/n-MoS_2_ monolayer devices, n-Si nanowire/n-MoS_2_ monolayer devices, n-MoS_2_ monolayer metal/semiconductor/metal (MSM) photoconductors, and p-Si nanowire/n-MoS_2_ multilayer devices. [157] The transient response is shown in Figure 12l. For p-Si nanowire/n-MoS_2_ monolayer devices, n-Si nanowire/n-MoS_2_ monolayer devices and MSM photoconductors devices, a response/recovery speed of 110 μs was estimated at instrumental resolution of 12 μs. When the instrumental resolution was increased to 200 kHz, the response and recovery times were estimated as 1.4/1.6 μs respectively. For a multilayer MoS_2_/Si nanowire, the response/recovery times were estimated as 0.7/1.1 μs respectively. Their findings suggest that the response time depends externally on the mobility of silicon nanowire which is of several orders higher than that of MoS_2_ and strong electric field created as a result of the depletion width at the interface [157]. Similarly, Wang et al. fabricated Pd-single layer MoS_2_ Schottky junction on SiO_2_/Si substrate [158]. A responsivity of 0.88 A/W and a response/recovery speed of 24.2/24.5 ms respectively were estimated when the device was illuminated with a 425 nm radiation [158]. Wang et al. developed a MoS_2_/CdTe p–n hetero-junction on Si/SiO_2_ substrate with device schematic shown in Figure 12m for broadband response up to 1700 nm, a responsivity of 36.6 mA/W and a response/recovery speed of 43.7/82.1 μs at a pulsed light illumination of 780 nm radiation was reported [159]. The transient response is shown in Figure 12n. Here, the carrier mobility of CdTe is of order 10^3^ cm^2^/Vs [160,161,162,163,164], which is several orders higher than that of MoS_2_^−^ and as expected, the hetero-structure has a high response time. Hao et al. developed MoS_2_/SiO_2_/Si p-i-n junction device structure [165]. The device was illuminated with 650 nm radiation and the response/recovery times were estimated as 16.2/160.5 μs respectively. As expected, the response time in this p-i-n device structure is controlled by the higher mobility of Si.

Tang et al. developed MoS_2_ nano-sheet photodetectors with ultrafast response on SiO_2_/Si substrate [166]. The device was illuminated with 532 radiation, a responsivity of 59 A/W and a response time of 42 μs were reported [166]. The authors fabricated the device with metals of different work functions such as Pd, Cr, Au and Ti. The fast response time can be attributed to the presence of a strong electric field at the interface between MoS_2_ and the metal contacts that forms a Schottky junction. Oliva et al. developed van der Waals MoS_2_/VO_2_ hetero-junction on SiO_2_/Si substrate for photodetector applications [167]. The device was illuminated with a 500/650 nm radiation, a maximum photoresponsivity of 1.25 A/W at 550 nm and a response time of 3.5 ms were reported [167]. By considering the mobility of VO_2_ reported between 0.07–2.65 cm^2^/Vs [168,169,170,171,172,173]. This analysis suggests that the mobility of VO_2_ has little effect on response/recovery speed of MoS_2_/VO_2_ hetero-structure, if any, it could be due a strong built-in electric field at the interface of the constituent semiconductors. Liu et al. fabricated a MoS_2_/GaN NW p–n junction on SiO_2_/Si substrate for photodetector applications [174]. The device was illuminated with a 550 nm radiation, a responsivity of 443.3 A/W and a response/recovery speed of 5 ms were reported [174]. The response time is 20 times faster probably due to anisotropic dependence of mobility for GaN nano-wire [175]. Lei et al. fabricated a MoS_2_/black phosphorous (BP) heterostructure photodetector [176] with responsivity of ~153.4 mA/W under 1550 nm illumination with rise and decay times of 15/70 μs. The fast response times can be attributed to the high mobility of BP. The detailed response/recovery speed and their device structures are summarized in Table 2.

**Figure 13:**
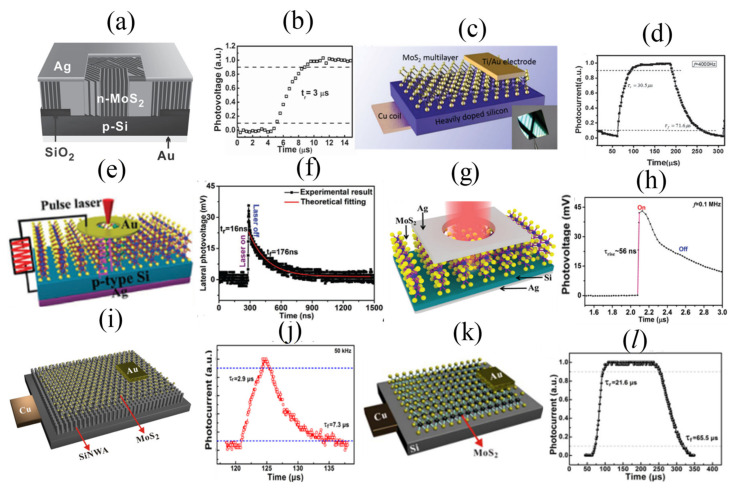
(**a**,**b**) Schematic illustration of the MoS_2_/Si hetero-junction-based photodetector, enlarged rise edge of the photoresponse curve. Reproduced with permission from reference [178]. (**c**,**d**) Schematic representation of the vertical multilayered MoS_2_/Si hetero-junction, magnified and normalized plots of one response cycle. Reproduced with permission from reference [179]. (**e**,**f**) Schematic of V-MoS_2_/Si hetero-junction, time-dependent lateral photovoltage for one pulse illumination (pulse width of 100 fs), Reproduced with permission from reference [180]. (**g**,**h**) Schematic illustration of the photoresponse of the V-MoS_2_/Si hetero-junction device, Time-dependent photovoltage at frequencies of 0.1 MHz (100 fs-pulse-width pulsed laser), Reproduced with permission from reference [181]. (**i**,**j**) Schematic diagram of a MoS_2_/Si NWA hetero-junction device fabrication. Rising and falling edges for estimating rise time (*τ*_r_) and the fall time (*τ*_f_) at 50 kHz, Reproduced with permission from reference [183]. (**k**,**l**) Schematic illustration of a MoS_2_/Si hetero-junction device, Rising and falling edges for estimating the rise time (*τ*_r_) and the fall time (*τ*_f_). Reproduced with permission from reference [184].

We now shift our attention to hetero-structure devices based on MoS_2_ that are fabricated with one of the semiconductors taken as p/n-doped Si. The carrier mobility of n or p-doped Si ranges between 500–1500 cm^2^/Vs [59]. Such devices are expected to have a fast response/recovery speeds in the order of few μs or ns and for defectless devices, it should approach ps limit. For example, Wang et al. developed polycrystalline n-MoS_2_ of 150 nm thick on p-Si substrate with device structure shown in Figure 13a via magnetron sputtering for self-driven visible-near infrared photodetection [178]. The device was illuminated with a 808 nm radiation, a responsivity of 300 mA/W and ultra-fast response/recovery speed of 3/40 μs were reported [178]. The transient response for half-cycle is shown in Figure 13b. The high speed and self-driven response was partly attributed to the existence of a strong build-in electric field at the MoS_2_ and Si interface [178]. Zhang et al. developed n-MoS_2_/n-Si vertical multilayered hetero-junction shown in the schematic of Figure 13c for high-speed visible-near-infrared photodetectors by two step thermolysis [179]. The device was irradiated with light of wave-length 650 nm radiation, and a responsivity of 11.9 A/W and a response/recovery speed of 30.5/71.6 μs were reported [179]. The transient response is shown in Figure 13d. The high-speed response was attributed to good quality synthesized MoS_2_ films and the reliable contact quality at the interface. Cong et al. developed vertically standing few layer MoS_2_/p-Si hetero-junction photodetector with schematic shown in Figure 13e [180]. The device was illuminated by lasers of different wavelengths (405, 532, 671, 808, and 980 nm) as the illumination source. The 532 nm laser was used for response/recovery time measurements. An ultrafast response/recovery times of 16/176 ns respectively were estimated. The transient response for once cycle is shown in Figure 13f. The ultrafast response times can be attributed to the excellent quality of V-MoS_2_/Si hetero-junction with strong light absorption and quick carrier transport speed in the unique vertically oriented few-layer MoS_2_ nano-sheets and large built-in electric field at the interface of V-MoS_2_ and Si [180]. Qiao et al. developed a vertically layered MoS_2_/Si hetero-junction with device structure shown in Figure 13g for an ultrahigh and ultrafast broad band photoresponse from 350–1100 nm [181]. A responsivity of up to 908.2 mA/W and a response/recovery speed estimated as ~56/825 ns were reported. The transient response for one cycle is shown in Figure 13h. Kim et al. developed a high-performing MoS_2_-embedded Si photodetector with MoS_2_/n-Si/p-Si device architecture [182]. An ultra-fast fast response/recovery speed of 33/30 µs respectively were measured when the device was illuminated with a wavelength of around 515–520 nm. Wu et al. developed MoS_2_/Si nanowire array hetero-junction shown in Figure 13i [183]. The device was illuminated with a 650 nm radiation, a responsivity of 53.5 A/W and a fast response/recovery speed of 2.9/7.3 μs respectively were reported [183]. The transient response is shown in Figure 13j. The same authors developed MoS_2_/Si hetero-junction shown in Figure 13k for broadband photodetectors from deep ultraviolet to near infrared [184]. The device was illuminated with a 780 nm radiation, a responsivity of 23.1 A/W and a response/recovery speed of 21.6/65.5 μs were reported [184]. The transient response is shown in Figure 13l. Dhyani et al. developed n-MoS_2_/porous silicon device structure [185]. The device was illuminated with light of wavelength between 550 and 850 nm, a responsivity of 9 A/W and a fast response/recovery speed of 9/7 μs were reported [185]. Their findings were compared with planar Si-MoS_2_ hetero-junction which had a response/recovery speed of 35/8.1 μs respectively. Their analysis clearly suggest that charge collection efficiency in the porous hetero-structure is quite high compared to the planar device [185]. The same authors (Dhyani et al.) developed a high-speed Si/MoS_2_ p–n hetero-junction photodetector [186]. The device was illuminated with a 580 nm radiation, a responsivity of 8.75 A/W at 580 nm and a response/recovery speed of 10/19 μs was reported [186]. Kim et al. fabricated MoS_2_ layers on p-Si substrate by sputtering method for efficient photoelectric application [187]. The device was illuminated with a 455 nm radiation, a responsivity of 0.03 A/W and a response/recovery speed of 38.78/43.07 μs were reported [187]. Recently, Guo et al. [188] used a broadband photodetector of MoS_2_/p^+^-Si with a responsivity of ~0.746 A/W under 808 nm illumination with rise and decay times of ~178/198 μs. The fast response times can be attributed to the inbuilt potential at the interface and also to the comparatively high mobility of p^+^-Si. The details are summarized in Table 3.

**Figure 14:**
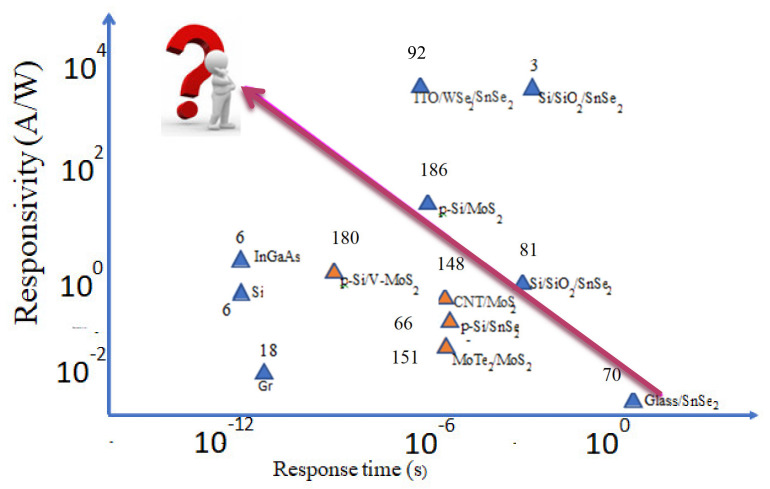
Reported response time and responsivities in comparison to Graphene, Si and InGaAs photodetectors.

As a benchmark, we compare the response time of graphene, commercial Si and InGaAs photodiodes with those of SnSe_2_, MoS_2_, and their related hetero-structures as summarized in Figure 14. Mittendorff et al. fabricated ultrafast graphene-based broadband THz detector on Si/SiO_2_ substrate. The response time was estimated as 50–100 ps in the wavelength range from 30 μm to 220 μm [18]. Commercial Si and InGaAs photodiodes are reported to have a response time of about 50 ps [6].

## 6. Summary and Perspective

We have reviewed and analyzed the factors that influence the response time of photodetectors based on 2D SnSe_2_, MoS_2_ and their related hetero-structures in relation to their constituent carrier mobility, built-in electric field at the interface and compared the findings to graphene and conventional materials of Si and InGaAs. Our systematic analysis suggests that the visible and NIR responsivity of 2D SnSe_2_ and MoS_2_ on insulating substrates (substrates with very low carrier mobility) is greater than those of graphene, Si and InGaAs, their response time is in the order of few milliseconds to seconds and hinder their applications in devices that require high speed. Other efforts have been made by developing hetero-structures with materials/substrates of higher mobility, including graphene and Si, and this has resulted in an increase in response time to a few μs, maintaining the overall performance of the device. We would like to mention here that this review has focused on 2D SnSe_2_, MoS_2_, and their related hetero-structures and there are a variety of other visible and NIR-active 2D-materials and their related hetero-structures which we have not discussed in this article but which have similar advantages and draw backs to those of SnSe_2_ and MoS_2_. On a challenging note, the ultrahigh responsivity reported here requires the application of a certain bias voltage which would eventually increase the operational cost of the devices. To minimize this would require developing hetero-structures or Schottky diodes with a strong built-in electric field although it saturates within certain region of the interface. This requires identifying materials with a large difference in the work-functions. In realizing a response time that matches that of graphene, Si, and InGaAs, keeping the overall performance of the device remains a grand challenge. In addition, identifying materials with high visible transparency, higher mobility, a high infrared optical absorption coefficient, and developing hetero-structures with low defect states at the interface also remains challenging. To harness the advantages of these 2D-layered materials and realize the desired goals in potential device applications, incorporating them with other materials of higher mobility with a large difference in the work-functions and minimizing the interfacial defect states seems to be the important way forward for the further development of these class of photodetectors.

## Figures and Tables

**Table 1:** Wavelength range, responsivity, response/recovery speed of SnSe_2_, and its related hetero-structures.

Device Structure	Detection Wavelength (nm)	Responsivity (A/W)	Response/Recovery Time	Ref
SLG/SnSe_2_-Bulk	1064	~2 × 10^−3^	7.76 s/2.5 s	[71]
SLG/SnSe_2_/PEDOT:PSS–Bulk	1064	~1.4–2.6 × 10^−6^	1.33 s/1.22 s	[5]
p-Si/n-SnSe_2_-Bulk	1064	~0.12	57 ± 25/34 ± 15 μs	[66]
Bi-layer (BL) SnSe_2_/SiO_2_/Si	633	~0.5	2.1 ± 0.3/3.2 ± 0.2 ms	[81]
SnSe_2_ flakes/SiO_2_/Si	530	~1.1 × 10^3^	14.5/8.1 ms	[3]
SnSe_2_/SiO_2_/Si	543	~0.48	17/45 μs	[82]
ITO/WSe_2_/SnSe_2_/SiO_2_/Si	785	~1100	10 μs	[92]
SnSe_2_/MoS_2_/SiO_2_/Si	500	~9.1 × 10^3^	0.2/0.6 s	[83]
ITO/SnSe_2_	white light	-	310/340 ms	[84]
SnSe_2_/SLG	1064	~0.8 × 10^−3^	276/332 ms	[105]

**Table 2:** Wavelength range, responsivity, response/recovery speed of MoS_2_ on SiO_2_/Si substrate and its related hetero-structures.

Device Structure	Detection Wavelength (nm)	Responsivity (A/W)	Response/Recovery Time	Ref
Single layer MoS_2_	450	~7.5 × 10^−^^3^	50/50 ms	[107]
P(VDF-TrFE) driven MoS_2_	635	~2570	1.8/2.0 ms	[114]
MoS_2_/CsPbBr_3_	442	~4.4	0.72/1.01 ms	[117]
CsPbBr_3_/MoS_2_	442	~13.1	2.5/1.8 ms	[119]
ZnPc treated MoS_2_ with Al_2_O_3_ passivation layer	532	~430	100 ms	[109]
CH_3_NH_3_PbI_3_/MoS_2_ with r-GO as HTL	660	~1.08 × 10^4^	<45 ms	[110]
GaSe/MoS_2_	532	~ 3	50 ms	[111]
BL MoS_2_	400–670	~50–700	174/166 ms	[112]
CdSe QDs/BL MoS_2_			159/172 ms	[109]
Core/shell CdSe/ZnS QDs/BL MoS_2_			185/172 ms	[109]
MoS_2_/h-BN/graphene	405	~180	0.23/0.25 s	[113]
MoS_2_/MoSe_2_	633	~350	10 ms	[133]
MoS_2_/CuO	570	~157.6	~34.6/51.9 ms	[138]
MoS_2_/Graphene QDs	405	~10^4^	0.07/1.23 s	[142]
MA_3_Bi_2_Br_9_ passivated few-layer MoS_2_	530	~112	0.3/0.3 ms	[145]
3D r-GO-MoS_2_/pyramid Si	808	~21.8	2.8/46.6 ms	[146]
Pd-MoS_2_/Si	950	~0.654	2.1/173.8 μs	[147]
CNT-MoS_2_ p-n junction	650	~0.1	~15/15 μs	[148]
MoTe_2_/MoS_2_	637	0.046	60/25 μs	[151]
Si NW-n-MoS_2_	500	-	1.4/1.6 μs	[157]
Pd-Single layer MoS_2_	425	~0.88	24.2/24.5 ms	[158]
MoS_2_-CdTe	780	~0.0366	43.7/82.1 μs	[159]
MoS_2_/SiO_2_/Si p-i-n junction	650	-	16.2/160.5 μs	[165]
MoS_2_ nanosheet	532	~59	42 ms	[166]
MoS_2_/VO_2_	550	~1.25	3.5 ms	[167]
MoS_2_-GaN NW p-n junction	550	~443.3	5 ms	[174]
MoS_2_/PbS	400–1500	6 × 10^5^	0.3–0.4 s	[53]
Graphene/MoS_2_	635	5 × 10^8^	-	[54]
WS_2_/MoS_2_	532	2340	_	[177]
MoS_2_/BP	1550	~0.153	15/70 μs	[176]

**Table 3:** Wavelength range, responsivity, response/recovery speed of MoS_2_ on p/n doped Si substrate.

Device Structure	Detection Wavelength (nm)	Responsivity (A/W)	Response/Recovery time	Ref
n-MoS_2_/p-Si	808	~0.3	3/40 μs	[178]
n-MoS_2_/n-Si	650	~11.9	30.5/71.6 μs	[179]
V-MoS_2_/p-Si	532	-	16/176 ns	[180]
MoS_2_/Si	808	~0.9082	~56/825 ns	[181]
MoS_2_/n-Si/p-Si	515–520	-	~33/30 μs	[182]
MoS_2/_Si NW array	650	~53.5	2.9/7.3 μs	[183]
MoS_2_/Si	780	~23.1	21.6/65.5 μs	[184]
n-MoS_2_/p-porous Si	550	~9	9/7 μs	[185]
Si/MoS_2_ p-n junction	580	~8.75	10/19 μs	[186]
MoS_2_/p-Si	455	~0.03	38.78/43.07 μs	[187]
MoS_2_/p^+^-Si	808	~0.746	~178/198 μs	[188]

## References

[1] Huo N., Konstantatos G. (2018). Recent Progress and Future Prospects of 2D-Based Photodetectors. Adv. Mater..

[2] Liu Y., Wang J., Huang H., Yun Y., Meng D., Yang Q., Zhai X., Fu Z., Knize R.J., Lu Y. (2017). Ferroelectric Polarization-Assisted Sensitive and High-Power Photodetector in Broad Ultraviolet-to-Visible Range. Adv. Opt. Mater..

[3] Zhou X., Gan L., Tian W., Zhang Q., Jin S., Li H., Bando Y., Golberg D., Zhai T. (2015). Ultrathin SnSe2 Flakes Grown by Chemical Vapor Deposition for High-Performance Photodetectors. Adv. Mater..

[4] Xie C., Mak C., Tao X., Yan F. (2017). Photodetectors Based on Two-Dimensional Layered Materials Beyond Graphene. Adv. Funct. Mater..

[5] Mukhokosi E.P., Krupanidhi S.B., Nanda K.K. (2018). An Extrinsic Approach Toward Achieving Fast Response and Self-Powered Photodetector. Phys. Status Solidi A.

[6] Buscema M., Island J.O., Groenendijk D.J., Blanter S.I., Steele G.A., van der Zant H.S.J., Castellanos-Gomez A. (2015). Photocurrent generation with two-dimensional van der Waals semiconductors. Chem. Soc. Rev..

[7] Huynh W.U., Dittmer J.J., Teclemariam N., Milliron D., Alivisatos A.P., Barnham K. (2003). Charge transport in hybrid nanorod-polymer composite photovoltaic cells. Phys. Rev. B.

[8] Yu Y., Zhang Z., Yin X., Kvit A., Liao Q., Kang Z., Yan X., Zhang Y., Wang X. (2017). Enhanced photoelectrochemical efficiency and stability using a conformal TiO_2_ film on a black silicon photoanode. Nat. Energy.

[9] Low T., Engel M., Steiner M., Avouris P. (2014). Origin of photoresponse in black phosphorus phototransistors. Phys. Rev. B Condens. Matter Mater. Phys..

[10] Mueller T., Xia F., Freitag M., Tsang J., Avouris P. (2009). Role of contacts in graphene transistors: A scanning photocurrent study. Phys. Rev. B Condens. Matter Mater. Phys..

[11] Lin P., Yan X., Zhang Z., Shen Y., Zhao Y., Bai Z., Zhang Y. (2013). Self-powered UV photosensor based on PEDOT:PSS/ZnO micro/nanowire with strain-modulated photoresponse. ACS Appl. Mater. Interfaces.

[12] Zhang Y., Yan X., Yang Y., Huang Y., Liao Q., Qi J. (2012). Scanning probe study on the piezotronic effect in ZnO nanomaterials and nanodevices. Adv. Mater..

[13] Zhang Z., Liao Q., Yu Y., Wang X., Zhang Y. (2014). Enhanced photoresponse of ZnO nanorods-based self-powered photodetector by piezotronic interface engineering. Nano Energy.

[14] Zhang Y., Yang Y., Gu Y., Yan X., Liao Q., Li P., Zhang Z., Wang Z. (2015). Performance and service behavior in 1-D nanostructured energy conversion devices. Nano Energy.

[15] Geng D., Yang H.Y. (2018). Recent Advances in Growth of Novel 2D Materials: Beyond Graphene and Transition Metal Dichalcogenides. Adv. Mater..

[16] Novoselov K.S., Geim A.K., Morozov S.V., Jiang D., Zhang Y., Dubonos S.V., Grigorieva I.V., Firsov A.A. (2004). Electric Field Effect in Atomically Thin Carbon Films. Sci. Vol..

[17] Novoselov K.S., Fal’ko V.I., Colombo L., Gellert P.R., Schwab M.G., Kim K. (2012). A roadmap for graphene. Nature.

[18] Mittendorff M., Winnerl S., Kamann J., Eroms J., Weiss D., Schneider H., Helm M. (2013). Ultrafast graphene-based broadband THz detector. Appl. Phys. Lett..

[19] Xia F., Mueller T., Lin Y.M., Valdes-Garcia A., Avouris P. (2009). Ultrafast graphene photodetector. Nat. Nanotechnol..

[20] Bao Q., Loh K.P. (2012). Graphene photonics, plasmonics, and broadband optoelectronic devices. ACS Nano.

[21] Avouris P. (2010). Graphene: Electronic and photonic properties and devices. Nano Lett..

[22] Bonaccorso F., Sun Z., Hasan T., Ferrari A.C. (2010). Graphene photonics and optoelectronics. Nat. Photonics.

[23] Mueller T., Xia F., Avouris P. (2010). Graphene photodetectors for high-speed optical communications. Nat. Photonics.

[24] Eng P.C., Song S., Ping B. (2015). State-of-the-art photodetectors for optoelectronic integration at telecommunication wavelength. Nanophotonics.

[25] Nguyen B.H., Nguyen V.H. (2016). Advances in graphene-based optoelectronics, plasmonics and photonics. Adv. Nat. Sci. Nanosci. Nanotechnol..

[26] Feng W., Zheng W., Chen X., Liu G., Cao W., Hu P. (2015). Solid-state reaction synthesis of a InSe/CuInSe_2_ lateral p-n heterojunction and application in high performance optoelectronic devices. Chem. Mater..

[27] Sun Z., Chang H. (2014). Graphene and Graphene-like Two-Dimensional Materials in Photodetection: Mechanisms and Methodology. ACS Nano.

[28] Zhang B.Y., Liu T., Meng B., Li X., Liang G., Hu X., Wang Q.J. (2013). Broadband high photoresponse from pure monolayer graphene photodetector. Nat. Commun..

[29] De Fazio D., Goykhman I., Yoon D., Bruna M., Eiden A., Milana S., Sassi U., Barbone M., Dumcenco D., Marinov K. (2016). High Responsivity, Large-Area Graphene/MoS_2_ Flexible Photodetectors. ACS Nano.

[30] Goykhman I., Sassi U., Desiatov B., Mazurski N., Milana S., de Fazio D., Eiden A., Khurgin J., Shappir J., Levy U. (2016). On-Chip Integrated, Silicon-Graphene Plasmonic Schottky Photodetector with High Responsivity and Avalanche Photogain. Nano Lett..

[31] Late D.J., Huang Y.K., Liu B., Acharya J., Shirodkar S.N., Luo J., Yan A., Charles D., Waghmare U.V., Dravid V.P. (2013). Sensing behavior of atomically thin-layered MoS_2_ transistors. ACS Nano.

[32] Lopez-Sanchez O., Lembke D., Kayci M., Radenovic A., Kis A. (2013). Ultrasensitive photodetectors based on monolayer MoS_2_. Nat. Nanotechnol..

[33] Ramakrishna Matte H.S.S., Gomathi A., Manna A.K., Late D.J., Datta R., Pati S.K., Rao C.N.R. (2010). MoS_2_ and WS_2_ analogues of graphene. Angew. Chem. Int. Ed..

[34] Mak K.F., Lee C., Hone J., Shan J., Heinz T.F. (2010). Atomically thin MoS_2_: A new direct-gap semiconductor. Phys. Rev. Lett..

[35] Choi W., Cho M.Y., Konar A., Lee J.H., Cha G.B., Hong S.C., Kim S., Kim J., Jena D., Joo J. (2012). High-detectivity multilayer MoS_2_ phototransistors with spectral response from ultraviolet to infrared. Adv. Mater..

[36] Gong C., Huang C., Miller J., Cheng L., Hao Y., Cobden D., Kim J., Ruoff R.S., Wallace R.M., Cho K. (2013). Metal Contacts on Physical Vapor Deposited Monolayer MoS_2_. ACS Nano.

[37] Dai J., Zeng X.C. (2014). Bilayer phosphorene: Effect of stacking order on bandgap and its potential applications in thin-film solar cells. J. Phys. Chem. Lett..

[38] Chang Y., Zhang O.W., Zhu O.Y., Han Y., Pu J., Chang J., Hsu W. (2014). Monolayer MoSe_2_ Grown by Chemical Vapor Deposition for Fast Photodetection. ACS Nano.

[39] Perea-Lõpez N., Elías A.L., Berkdemir A., Castro-Beltran A., Gutiérrez H.R., Feng S., Lv R., Hayashi T., Lõpez-Urías F., Ghosh S. (2013). Photosensor device based on few-layered WS_2_ films. Adv. Funct. Mater..

[40] Enyashin A.N., Yadgarov L., Houben L., Popov I., Weidenbach M., Tenne R., Bar-Sadan M., Seifert G. (2011). New Route for Stabilization of 1T-WS_2_ and MoS_2_ Phases. J. Phys. Chem. C.

[41] Gutiérrez H.R., Perea-López N., Elías A.L., Berkdemir A., Wang B., Lv R., López-Urías F., Crespi V.H., Terrones H., Terrones M. (2013). Extraordinary room-temperature photoluminescence in triangular WS_2_ monolayers. Nano Lett..

[42] Xia J., Huang X., Liu L.Z., Wang M., Wang L., Huang B., Zhu D.D., Li J.J., Gu C.Z., Meng X.M. (2014). CVD synthesis of large-area, highly crystalline MoSe_2_ atomic layers on diverse substrates and application to photodetectors. Nanoscale.

[43] Huang C., Wu S., Sanchez A.M., Peters J.J.P., Beanland R., Ross J.S., Rivera P., Yao W., Cobden D.H., Xu X. (2014). Lateral heterojunctions within monolayer MoSe_2_–WSe_2_ semiconductors. Nat. Mater..

[44] Huo N., Yang S., Wei Z., Li S.-S., Xia J.-B., Li J. (2015). Photoresponsive and Gas Sensing Field-Effect Transistors based on Multilayer WS_2_ Nanoflakes. Sci. Rep..

[45] Feng W., Zhou X., Tian W.Q., Zheng W., Hu P. (2015). Performance improvement of multilayer InSe transistors with optimized metal contacts. Phys. Chem. Chem. Phys..

[46] Tamalampudi S.R., Lu Y.Y., Kumar U.R., Sankar R., Liao C.D., Moorthy B.K., Cheng C.H., Chou F.C., Chen Y.T. (2014). High performance and bendable few-layered InSe photodetectors with broad spectral response. Nano Lett..

[47] Feng W., Zheng W., Cao W., Hu P. (2014). Back Gated Multilayer InSe Transistors with Enhanced Carrier Mobilities via the Suppression of Carrier Scattering from a Dielectric Interface. Adv. Mater..

[48] Late D.J., Liu B., Luo J., Yan A., Matte H.S.S.R., Grayson M., Rao C.N.R., Dravid V.P. (2012). GaS and GaSe ultrathin layer transistors. Adv. Mater..

[49] Zhou Y., Zhou Y., Nie Y., Liu Y., Yan K., Hong J., Jin C., Yin J., Liu Z., Peng H. (2014). Epitaxy and Photoresponse of Two-Dimensional GaSe Crystals on Flexible Transparent Mica Sheets. ACS Nano.

[50] Jacobs-Gedrim R.B., Shanmugam M., Jain N., Durcan C.A., Murphy M.T., Murray T.M., Matyi R.J., Moore R.L., Yu B. (2014). Extraordinary Photoresponse in Two-Dimensional In_2_Se_3_ Nanosheets. ACS Nano.

[51] Tian H., Chin M.L., Najmaei S., Guo Q., Xia F., Wang H., Dubey M. (2016). Optoelectronic devices based on two-dimensional transition metal dichalcogenides. Nano Res..

[52] Wang F., Wang Z., Yin L., Cheng R., Wang J., Wen Y., Shifa T.A., Wang F., Zhang Y., Zhan X. (2018). 2D library beyond graphene and transition metal dichalcogenides: A focus on photodetection. Chem. Soc. Rev..

[53] Kufer D., Nikitskiy I., Lasanta T., Navickaite G., Koppens F.H.L., Konstantatos G. (2015). Hybrid 2D-0D MoS_2_-PbS quantum dot photodetectors. Adv. Mater..

[54] Roy K., Padmanabhan M., Goswami S., Sai T.P., Ramalingam G., Raghavan S., Ghosh A. (2013). Graphene-MoS_2_ hybrid structures for multifunctional photoresponsive memory devices. Nat. Nanotechnol..

[55] Wang Z.L., Wu W. (2014). Piezotronics and piezo-phototronics: Fundamentals and applications. Natl. Sci. Rev..

[56] Wang Z.L. (2012). Progress in piezotronics and piezo-phototronics. Adv. Mater..

[57] Wang Z.L. (2012). Preface to the special section on piezotronics. Adv. Mater..

[58] Xia W., Dai L., Yu P., Tong X., Song W., Zhang G., Wang Z.M. (2017). Recent Progress in Van Der Waals Heterojunctions. Nanoscale.

[59] Sze S.M., Ng K.K. (2010). Physics of Semiconductor Devices.

[60] Gawȩda S., Kowalik R., Kwolek P., MacYk W., Mech J., Oszajca M., Podborska A., Szaciłowski K. (2011). Nanoscale digital devices based on the photoelectrochemical photocurrent switching effect: Preparation, properties and applications. Isr. J. Chem..

[61] Nandjou F., Haussener S. (2017). Degradation in photoelectrochemical devices: Review with an illustrative case study. J. Phys. D Appl. Phys..

[62] Pessoa R.S., Fraga M.A., Santos L.V., Massi M., Maciel H.S. (2015). Nanostructured thin films based on TiO_2_ and/or SiC for use in photoelectrochemical cells: A review of the material characteristics, synthesis and recent applications. Mater. Sci. Semicond. Process..

[63] Szaciłowski K., Macyk W. (2007). Photoelectrochemical photocurrent switching effect: A new platform for molecular logic devices. Chimia (Aarau).

[64] Serna M.I., Hasan S.M.N., Nam S., El Bouanani L., Moreno S., Choi H., Alshareef H.N., Minary-Jolandan M., Quevedo-Lopez M.A. (2018). Low-Temperature Deposition of Layered SnSe_2_ for Heterojunction Diodes. Adv. Mater. Interfaces.

[65] Shimada T., Ohuchi F.S., Parkinson B.A. (1994). Work Function and Photothreshold of Layered Metal Dichalcogenides. Jpn. J. Appl. Phys..

[66] Mukhokosi E.P., Roul B., Krupanidhi S.B., Nanda K.K. (2019). Towards fast and highly responsive SnSe_2_ based photodiode by exploiting the mobility of the counter semiconductor. ACS Appl. Mater. Interfaces.

[67] Xu X., Gabor N.M., Alden J.S., van der Zande A.M., McEuen P.L. (2010). Photo-thermoelectric effect at a graphene interface junction. Nano Lett..

[68] Richards P.L. (1994). Bolometers for infrared and millimeter waves. J. Appl. Phys..

[69] Koppens F.H.L., Mueller T., Avouris P., Ferrari A.C., Vitiello M.S., Polini M. (2014). Photodetectors based on graphene, other two-dimensional materials and hybrid systems. Nat. Nanotechnol..

[70] Huang Y., Xu K., Wang Z.X., Shifa T.A., Wang Q.S., Wang F., Jiang C., He J. (2015). Designing the shape evolution of SnSe_2_ nanosheets and their optoelectronic properties. Nanoscale.

[71] Mukhokosi E.P., Krupanidhi S.B., Nanda K.K. (2017). Band Gap Engineering of Hexagonal SnSe_2_ Nanostructured Thin Films for Infra-Red Photodetection. Sci. Rep..

[72] Gonzalez J.M., Oleynik I.I. (2016). Layer-dependent properties of SnS_2_ and SnSe_2_ novel two-dimensional materials. Phys. Rev. B.

[73] Schlaf R., Pettenkofer C., Jaegermann W. (1999). Band lineup of a SnS_2_/SnSe_2_/SnS_2_ semiconductor quantum well structure prepared by van der Waals epitaxy. J. Appl. Phys..

[74] Guo P., Luo Y.W., Jia Y. (2016). Tuning band gap and optical properties of SnX_2_ nanosheets: Hybrid functional studies. Mod. Phys. Lett. B.

[75] Evans B.L., Hazelwood R.A. (2002). Optical and electrical properties of SnSe_2_. J. Phys. D Appl. Phys..

[76] Lin Z., Mccreary A., Briggs N., Park Y.W., Jerng S., Jeon J.H., Roy S.B., Akbar K., Kim J. (2017). Molecular beam epitaxy of large-area SnSe_2_ with monolayer thickness fluctuation. 2D Mater..

[77] Barrios-Salgado E., Nair M.T.S., Nair P.K. (2016). Thin films of n-type SnSe_2_ produced from chemically deposited p-type SnSe. Thin Solid Films.

[78] Julien C., Eddrief M., Samaras I., Balkanski M. (1992). Optical and electrical characterizations of SnSe, SnS_2_ and SnSe_2_ single crystals. Mater. Sci. Eng. B.

[79] Amalraj L., Jayachandran M., Sanjeeviraja C. (2004). Preparation and characterization of tin diselenide thin film by spray pyrolysis technique. Mater. Res. Bull..

[80] Guo C., Tian Z., Xiao Y., Mi Q., Xue J. (2016). Field-effect transistors of high-mobility few-layer SnSe_2_. Appl. Phys. Lett..

[81] Yu P., Yu X., Lu W., Lin H., Sun L., Du K., Liu F., Fu W., Zeng Q., Shen Z. (2016). Fast Photoresponse from 1T Tin Diselenide Atomic Layers. Adv. Funct. Mater..

[82] Wu J., Hu Z., Jin Z., Lei S., Guo H., Chatterjee K., Zhang J., Yang Y., Li B., Liu Y. (2016). Spiral Growth of SnSe_2_ Crystals by Chemical Vapor Deposition. Adv. Mater. Interfaces.

[83] Zhou X., Zhou N., Li C., Song H., Zhang Q., Hu X., Gan L., Li H., Lü J., Luo J. (2017). Vertical heterostructures based on SnSe_2_/MoS_2_ for high performance photodetectors. 2D Mater..

[84] Pawbake A.S., Date A., Jadkar S.R., Late D.J. (2016). Temperature Dependent Raman Spectroscopy and Sensing Behavior of Few Layer SnSe_2_ Nanosheets. Chemistry Select.

[85] Podzorov V., Gershenson M.E., Kloc C., Zeis R., Bucher E. (2004). High-mobility field-effect transistors based on transition metal dichalcogenides High-mobility field-effect transistors based on transition metal dichalcogenides. Appl. Phys. Lett..

[86] Movva H.C.P., Rai A., Kang S., Kim K., Fallahazad B., Taniguchi T., Watanabe K., Tutuc E., Banerjee S.K. (2015). High-Mobility Holes in Dual-Gated WSe_2_ field effect transistors. ACS Nano.

[87] Chen C., Wu C., Pu J., Chiu M., Kumar P., Takenobu T., Li L. (2014). Hole mobility enhancement and p-doping in monolayer WSe_2_ by gold decoration Hole mobility enhancement and p-doping in monolayer WSe_2_ by gold decoration. 2D Mater..

[88] Fang H., Chuang S., Chang T.C., Takei K., Takahashi T., Javey A. (2012). High-Performance Single Layered WSe_2_ p-FETs with Chemically Doped Contacts. Nano Lett..

[89] Liu W., Kang J., Sarkar D., Khatami Y., Jena D., Banerjee K. (2013). Role of Metal Contacts in Designing High-Performance Monolayer n-Type WSe_2_ Field Effect Transistors. Nano Lett..

[90] Chuang H., Tan X., Ghimire N.J., Perera M.M., Chamlagain B., Cheng M.M., Yan J., Mandrus D., Toma D. (2014). High Mobility WSe 2 p- and n-Type Field-Effect Transistors Contacted by Highly Doped Graphene for Low-Resistance Contacts. Nano Lett..

[91] Wu Z., Luo Z., Shen Y., Zhao W., Wang W., Nan H., Guo X., Sun L., Wang X., You Y. (2016). Defects as a factor limiting carrier mobility in WSe_2_: A spectroscopic investigation. Nano Res..

[92] Murali K., Majumdar K. (2018). Self-Powered, Highly Sensitive, High-Speed Photodetection Using ITO/WSe_2_/SnSe_2_ Vertical Heterojunction. IEEE Trans. Electron. Devices.

[93] Lin M., Liu L., Lan Q., Tan X., Dhindsa K.S., Zeng P., Naik V.M. (2012). Mobility enhancement and highly efficient gating of monolayer MoS_2_ transistors with polymer electrolyte. J. Phys. D Appl. Phys..

[94] Zheng J., Yan X., Lu Z., Qiu H., Xu G., Zhou X. (2017). High-Mobility Multilayered MoS_2_ Flakes with Low Contact Resistance Grown by Chemical Vapor Deposition. Adv. Mater..

[95] Pradhan N.R., Rhodes D., Zhang Q., Talapatra S., Terrones M., Ajayan P.M. (2013). Intrinsic carrier mobility of multi-layered MoS_2_ field-effect transistors on SiO_2_. Appl. Phys. Lett..

[96] Bao W., Cai X., Kim D., Sridhara K., Fuhrer M.S. (2013). High mobility ambipolar MoS_2_ field-effect transistors: Substrate and dielectric effects High mobility ambipolar MoS_2_ field-effect transistors: Substrate and dielectric effects. Appl. Phys. Lett..

[97] Perera M.M., Lin M., Chuang H., Chamlagain B.P., Wang C., Tan X., Cheng M.M., Toma D. (2013). Improved Carrier Mobility in. ACS Nano.

[98] Chang C., Li H., Shi Y., Zhang H., Lai C., Li L. (2012). Growth of Large-Area and Highly Crystalline MoS_2_ Thin Layers on Insulating Substrates. Nano Lett..

[99] Cai Y., Zhang G., Zhang Y. (2014). Polarity-Reversed Robust Carrier Mobility in Monolayer MoS_2_ Nanoribbons. J. Am. Chem. Soc..

[100] Rai A., Movva H.C.P., Banerjee S.K., Roy A., Taneja D., Chowdhury S. (2018). Progress in Contact, Doping and Mobility Engineering of MoS_2_: An Atomically Thin. Crystals.

[101] Choi Y., Kim H., Yang J., Shin S.W., Um S.H., Lee S., Kang M.S., Cho J.H. (2018). Proton-Conductor-Gated MoS_2_ Transistors with Room Temperature Electron Mobility of >100 cm^2^ V^−1^ s^−1^. Chem. Mater..

[102] Shao P., Zhao H., Cao H., Wang X., Pang Y. (2016). Enhancement of carrier mobility in MoS_2_ field effect transistors by a SiO_2_ protective layer. Appl. Phys. Lett..

[103] Jariwala D., Sangwan V.K., Late D.J., Johns J.E., Dravid V.P., Marks T.J., Lauhon J., Hersam M.C. (2013). Band-like transport in high mobility unencapsulated single-layer MoS_2_ transistors Band-like transport in high mobility unencapsulated single-layer MoS_2_ transistors. Appl. Phys. Lett..

[104] Wu W., De D., Chang S., Wang Y., Peng H., Bao J. (2013). High mobility and high on/off ratio field-effect transistors based on chemical vapor deposited single-crystal MoS_2_ grains. Appl. Phys. Lett..

[105] Manoj K., Sanju R., Animesh P., Kuldeep S.G., Sudhir H., Preetam S., Singh V.N. (2020). Highly responsive, low-bias operated SnSe_2_ nanostructured thin film for trap-assisted NIR photodetector. J. Alloy. Compd..

[106] Radisavljevic B., Radenovic A., Brivio J., Giacometti V., Kis A. (2011). Single-layer MoS_2_ transistors. Nat. Nanotechnol..

[107] Yin Z., Li H., Li H., Jiang L., Shi Y., Sun Y., Lu G., Zhang Q., Chen X., Zhang H. (2012). Single-layer MoS_2_ phototransistors. ACS Nano.

[108] Song X., Liu X., Yu D., Huo C., Ji J., Li X., Zhang S., Zou Y., Zhu G., Wang Y. (2018). Boosting Two-Dimensional MoS_2_/CsPbBr_3_ Photodetectors via Enhanced Light Absorbance and Interfacial Carrier Separation. ACS Appl. Mater. Interfaces.

[109] Huang Y., Zhuge F., Hou J., Lv L., Luo P., Zhou N., Gan L., Zhai T. (2018). Van der Waals Coupled Organic Molecules with Monolayer MoS_2_ for Fast Response Photodetectors with Gate-Tunable Responsivity. ACS Nano.

[110] Peng Z.-Y., Xu J.-L., Zhang J.-Y., Gao X., Wang S.-D. (2018). Solution-Processed High-Performance Hybrid Photodetectors Enhanced by Perovskite/MoS_2_ Bulk Heterojunction. Adv. Mater. Interfaces.

[111] Islam A., Lee J., Feng P.X.-L. (2018). Atomic Layer GaSe/MoS_2_ van der Waals Heterostructure Photodiodes with Low Noise and Large Dynamic Range. ACS Photonics.

[112] Li M., Chen J.-S., Routh P.K., Zahl P., Nam C.-Y., Cotlet M. (2018). Distinct Optoelectronic Signatures for Charge Transfer and Energy Transfer in Quantum Dot-MoS_2_ Hybrid Photodetectors Revealed by Photocurrent Imaging Microscopy. Adv. Funct. Mater..

[113] Vu Q.A., Lee J.H., Nguyen V.L., Shin Y.S., Lim S.C., Lee K., Heo J., Park S., Kim K., Lee Y.H. (2017). Tuning Carrier Tunneling in van der Waals Heterostructures for Ultrahigh Detectivity. Nano Lett..

[114] Wang X., Wang P., Wang J., Hu W., Zhou X., Guo N., Huang H., Sun S., Shen H., Lin T. (2015). Ultrasensitive and Broadband MoS_2_ Photodetector Driven by Ferroelectrics. Adv. Mater..

[115] Zeng J., Li X., Wu Y., Yang D., Sun Z., Song Z., Wang H., Zeng H. (2018). Space-Confined Growth of CsPbBr_3_ Film Achieving Photodetectors with High Performance in All Figures of Merit. Adv. Funct. Mater..

[116] Yang Z., Wang M., Qiu H., Yao X., Lao X., Xu S., Lin Z., Sun L., Shao J. (2018). Engineering the Exciton Dissociation in Quantum-Confined 2D CsPbBr_3_ Nanosheet Films. Adv. Funct. Mater..

[117] Stoumpos C.C., Malliakas C.D., Peters J.A., Liu Z., Sebastian M., Im J., Chasapis T.C., Wibowo A.C., Chung D.Y., Freeman A.J. (2013). Crystal Growth of the Perovskite Semiconductor CsPbBr_3_: A New Material for High-Energy Radiation Detection. Cryst. Growth Des..

[118] Yang B., Zhang F., Chen J., Yang S., Xia X., Pullerits T. (2017). Ultrasensitive and Fast All-Inorganic Perovskite-Based Photodetector via Fast Carrier Diffusion. Adv. Mater..

[119] Huo C., Liu X., Wang Z., Song X., Zeng H. (2018). High-Performance Low-Voltage-Driven Phototransistors through CsPbBr_3_-2D Crystal van der Waals Heterojunctions. Adv. Opt. Mater..

[120] Pfuetzner S., Mickel C., Jankowski J., Hein M., Meiss J., Schuenemann C., Elschner C., Levin A.A., Rellinghaus B., Leo K. (2011). The influence of substrate heating on morphology and layer growth in C60: ZnPc bulk heterojunction solar cells. Org. Electron..

[121] Ohmori Y., Itoh E., Miyairi K. (2006). Photovoltaic properties of phthalocyanine based p–n diode evaporated onto titanium dioxide. Thin Solid Films.

[122] Maennig B., Pfeiffer M., Nollau A., Zhou X., Leo K., Simon P. (2001). Controlled p-type doping of polycrystalline and amorphous organic layers: Self-consistent description of conductivity and field-effect mobility by a microscopic percolation model. Phys. Rev. B.

[123] Wang L. (2017). The Nature of Electron Mobility in Hybrid Perovskite CH_3_ NH_3_ PbI_3_. Nano Lett..

[124] Dong Q., Fang Y., Shao Y., Qiu J., Cao L., Huang J. (2015). Electron-hole diffusion lengths >175 μm in solution-grown CH_3_NH_3_PbI_3_ single crystals. Science (80-).

[125] Milot R.L., Eperon G.E., Snaith H.J., Johnston M.B., Herz L.M. (2015). Temperature-Dependent Charge-Carrier Dynamics in CH_3_NH_3_PbI_3_ Perovskite Thin Films. Adv. Funct. Mater..

[126] Hasegawa M., Hirayama Y., Ohno Y., Maehashi K., Matsumoto K. (2014). Characterization of reduced graphene oxide field-effect transistor and its application to biosensor. Jpn. J. Appl. Phys..

[127] Yang J., Kim J., Shin H.S. (2012). Facile Method for rGO Field Effect Transistor: Selective Adsorption of rGO on SAM-Treated Gold Electrode by Electrostatic Attraction. Adv. Mater..

[128] Qasrawi A.F., Abdallah M.M.A. (2018). Performance of Ge-Sandwiched GaSe Layers. J. Electron. Mater..

[129] Tao W., Li J., Zhao Q., Yin Z., Zhang Y., Chen B., Xie Y., Jie W. (2018). High-Quality GaSe Single Crystal Grown by the Bridgman Method. Materials.

[130] Liu N., Zhou S., Gao N., Zhao J. (2018). Tuning Schottky barriers for monolayer GaSe FETs by exploiting a weak Fermi level pinning effect. Phys. Chem. Chem. Phys..

[131] Qasrawi A.F., Abdallah M.M.A. (2018). Effect of Au/Ge substrate on the properties of GaSe. Optik (Stuttg.).

[132] Tang L., Zhao Z., Yuan S., Yang T., Zhou B., Zhou H. (2018). Self-catalytic VLS growth one dimensional layered GaSe nanobelts for high performance photodetectors. J. Phys. Chem. Solids.

[133] Yang Y., Huo N., Li J. (2017). Gate modulated and enhanced optoelectronic performance of MoSe_2_ and CVD-grown MoS_2_ heterojunctions. RSC Adv..

[134] Lee H., Ahn J., Im S., Kim J., Choi W. (2018). High-Responsivity Multilayer MoSe_2_ Phototransistors with Fast Response Time. Sci. Rep..

[135] Larentis S., Fallahazad B., Tutuc E. (2012). Field-effect transistors and intrinsic mobility in ultra-thin MoSe_2_ layers. Appl. Phys. Lett..

[136] Wang X., Gong Y., Shi G., Chow W.L., Keyshar K., Ye G.R., Lou J., Liu Z., Ringe E., Tay B.K. (2014). Chemical Vapor Deposition Growth. ACS Nano.

[137] Chamlagain B., Li Q., Ghimire N.J., Chuang H., Perera M.M., Tu H., Xu Y., Pan M., Xaio D., Yan J. (2014). Mobility Improvement and Temperature Dependence in MoSe_2_ Field-Effect Transistors on Parylene-C. ACS Nano.

[138] Um D.S., Lee Y., Lim S., Park S., Lee H., Ko H. (2016). High-Performance MoS_2_/CuO Nanosheet-on-One-Dimensional Heterojunction Photodetectors. ACS Appl. Mater. Interfaces.

[139] Sanal K.C., Vikas L.S., Jayaraj M.K. (2014). Applied Surface Science Room temperature deposited transparent p-channel CuO thin film transistors. Appl. Surf. Sci..

[140] Shen Y., Guo M., Shao G. (2015). ScienceDirect Role of materials chemistry on the electrical/electronic properties of CuO thin films. Acta Mater..

[141] Sung S., Kim S., Jo K., Lee J., Kim J., Kim S., Chai H., Pearton S.J., Norton D.P., Heo Y. (2010). Fabrication of p-channel thin-film transistors using CuO active layers deposited at low temperature. Appl. Phys. Lett..

[142] Chen C., Qiao H., Lin S., Man Luk C., Liu Y., Xu Z., Song J., Xue Y., Li D., Yuan J. (2015). Highly responsive MoS_2_ photodetectors enhanced by graphene quantum dots. Sci. Rep..

[143] Li Y., Hu Y., Zhao Y., Shi G., Deng L., Hou Y., Qu L. (2011). An Electrochemical Avenue to Green-Luminescent Graphene Quantum Dots as Potential Electron-Acceptors for Photovoltaics. Adv. Mater..

[144] Bacon M., Bradley S.J., Nann T. (2014). Graphene Quantum Dots. Part. Part. Syst. Charact..

[145] He J., Yang Y., He Y., Ge C., Zhao Y., Gao L., Tang J. (2018). Low Noise and Fast Photoresponse of Few-Layered MoS_2_ Passivated by MA_3_Bi_2_Br_9_. ACS Photonics.

[146] Xiao P., Mao J., Ding K., Luo W., Hu W., Zhang X., Zhang X., Jie J. (2018). Solution-Processed 3D RGO–MoS_2_/Pyramid Si Heterojunction for Ultrahigh Detectivity and Ultra-Broadband Photodetection. Adv. Mater..

[147] Hao L.Z., Gao W., Liu Y.J., Liu Y.M., Han Z.D., Xue Q.Z., Zhu J. (2015). Self-powered broadband, high-detectivity and ultrafast photodetectors based on Pd-MoS_2_/Si heterojunctions. Phys. Chem. Chem. Phys..

[148] Jariwala D., Sangwan V.K., Wu C.-C., Prabhumirashi P.L., Geier M.L., Marks T.J., Lauhon L.J., Hersam M.C. (2013). Gate-tunable carbon nanotube-MoS_2_ heterojunction p-n diode. Proc. Natl. Acad. Sci. USA.

[149] Snow E.S., Campbell P.M., Ancona M.G., Novak J.P., Snow E.S., Campbell P.M., Ancona M.G. (2011). High-mobility carbon-nanotube thin-film transistors on a polymeric substrate High-mobility carbon-nanotube thin-film transistors on a polymeric substrate. Appl. Phys. Lett..

[150] Getty S.A., Cobas E., Fuhrer M.S. (2004). Extraordinary Mobility in Semiconducting Carbon Nanotubes. Nano Lett..

[151] Chen Y., Wang X., Wu G., Wang Z., Fang H., Lin T., Sun S., Shen H., Hu W., Wang J. (2018). High-Performance Photovoltaic Detector Based on MoTe_2_/MoS_2_ Van der Waals Heterostructure. Small.

[152] Keum D.H., Cho S., Kim J.H., Choe D., Sung H., Kan M., Kang H., Hwang J., Kim S.W., Yang H. (2015). Bandgap opening in few-layered monoclinic MoTe_2_. Nat. Phys..

[153] Mote R., Pradhan N.R., Rhodes D., Feng S., Xin Y., Memaran S., Moon B. (2014). Field-Effect Transistors Based on Few-Layered R-MoTe_2_. ACS Nano.

[154] Lin Y., Xu Y., Wang S., Li S., Yamamoto M. (2014). Ambipolar MoTe_2_ Transistors and Their Applications in Logic Circuits. Adv. Mater..

[155] Zhou L., Xu K., Zubair A., Liao A.D., Fang W., Ouyang F., Lee Y., Ueno K., Saito R., Dresselhaus M.S. (2015). Large-Area Synthesis of High-Quality Uniform Few-Layer MoTe_2_. J. Am. Chem. Soc..

[156] Cho S., Kim S., Kim J.H., Zhao J., Seok J., Keum D.H., Baik J., Choe D., Chang K.J., Suenaga K. (2015). Phase patterning for ohmic homojunction contact in MoTe_2_. Science (80-).

[157] Henning A., Sangwan V.K., Bergeron H., Balla I., Sun Z., Hersam M.C., Lauhon L.J. (2018). Charge Separation at Mixed-Dimensional Single and Multilayer MoS_2_/Silicon Nanowire Heterojunctions. ACS Appl. Mater. Interfaces.

[158] Wang X.-F., Zhao H.-M., Shen S.-H., Pang Y., Shao P.-Z., Li Y.-T., Deng N.-Q., Li Y.-X., Yang Y., Ren T.-L. (2016). High performance photodetector based on Pd-single layer MoS_2_ Schottky junction. Appl. Phys. Lett..

[159] Wang Y., Huang X., Wu D., Zhuo R., Wu E., Jia C., Shi Z., Xu T., Tian Y., Li X. (2018). A room-temperature near-infrared photodetector based on a MoS_2_/CdTe p-n heterojunction with a broadband response up to 1700 nm. J. Mater. Chem. C.

[160] Turkevych I., Grill R., Franc J., Belas E. (2002). High-temperature electron and hole mobility in CdTe. Semicond. Sci. Technol..

[161] Bicknell R.N., Giles N.C., Schetzina J.F., Bicknell R.N., Giles N.C., Schetzina J.F. (2003). Growth of high mobility n-type CdTe by photoassisted molecular beam epitaxy Growth of hijgh mobiUty n-type CdTe by photoassisted epitaxy. Appl. Phys. Lett..

[162] Martini C.C.M., Zanio G.O.K. (1970). Time of Flight Measurement of the Differential Negative Mobility in CdTe. Phys. Lett..

[163] Greene S.K., Singleton J. (1990). Fundamental Properties of High Mobility InSb-CdTe Heterojunctions. Surf. Sci..

[164] Sellin P.J., Davies A.W., Lohstroh A., Özsan M.E., Parkin J. (2005). Drift Mobility and Mobility-Lifetime Products in CdTe: Cl Grown by the Travelling Heater Method. IEEE Trans. Nucl. Sci..

[165] Hao L.Z., Liu Y.J., Han Z.D., Xu Z.J., Zhu J. (2018). Giant lateral photovoltaic effect in MoS_2_/SiO_2_/Si p-i-n junction. J. Alloys Compd..

[166] Tang W., Liu C., Wang L., Chen X., Luo M., Guo W., Wang S.W., Lu W. (2017). MoS_2_ nanosheet photodetectors with ultrafast response. Appl. Phys. Lett..

[167] Oliva N., Casu E.A., Yan C., Krammer A., Rosca T., Magrez A., Stolichnov I., Schueler A., Martin O.J.F., Ionescu A.M. (2017). Van der Waals MoS_2_/VO_2_ heterostructure junction with tunable rectifier behavior and efficient photoresponse. Sci. Rep..

[168] Fisher B. (1982). Electrical and Seebeck Effect Measurements in Nb. J. Phys. Chem. Solids.

[169] Goodenough J.B. (1971). The Two Components of the Crystallographic Transition in VO_2_. J. Solid State Chem..

[170] Maeng J., Kim T., Jo G., Lee T. (2008). Fabrication, structural and electrical characterization of VO_2_ nanowires. Mater. Res. Bull..

[171] Kwan C.C.Y., Griffiths C.H., Eastwood H.K. (2012). Transport and Structural Properties of VO_2_ Films. Appl. Phys. Lett..

[172] Fu D., Liu K., Tao T., Lo K., Cheng C., Liu B., Zhang R. (2013). Epitaxial VO_2_ thin films Comprehensive study of the metal-insulator transition in pulsed laser deposited epitaxial VO_2_ thin films. J. Appl. Phys..

[173] Kittiwatanakul S., Lu J., Wolf S.A. (2011). Transport Anisotropy of Epitaxial VO_2_ Films near the Metal—Semiconductor Transition. Appl. Phys. Express.

[174] Liu X., Yang X., Gao G., Yang Z., Liu H., Li Q., Lou Z., Shen G., Liao L., Pan C. (2016). Enhancing Photoresponsivity of Self-Aligned MoS_2_ Field-Effect Transistors by Piezo-Phototronic Effect from GaN Nanowires. ACS Nano.

[175] Pant R., Shetty A., Chandan G., Roul B., Nanda K.K. (2018). In-Plane Anisotropic Photoconduction in Nonpolar Epitaxial a-Plane GaN. ACS Appl. Mater. Interfaces.

[176] Ye L., Li H., Chen Z., Xu J. (2016). Near-Infrared Photodetector Based on MoS_2_/Black Phosphorous Heterojunction. ACS Photonics.

[177] Tan H., Xu W., Sheng Y., Lau C.S., Fan Y., Chen Q., Tweedie M., Wang X., Zhou Y., Warner J.H. (2017). Lateral Graphene-Contacted Vertically Stacked WS_2_/MoS_2_ Hybrid Photodetectors with Large Gain. Adv. Mater..

[178] Wang L., Jie J., Shao Z., Zhang Q., Zhang X., Wang Y., Sun Z., Lee S.T. (2015). MoS_2_/Si heterojunction with vertically standing layered structure for ultrafast, high-detectivity, self-driven visible-near infrared photodetectors. Adv. Funct. Mater..

[179] Zhang Y., Yu Y., Mi L., Wang H., Zhu Z., Wu Q., Zhang Y., Jiang Y. (2016). In Situ Fabrication of Vertical Multilayered MoS_2_/Si Homotype Heterojunction for High-Speed Visible-Near-Infrared Photodetectors. Small.

[180] Cong R., Qiao S., Liu J., Mi J., Yu W., Liang B., Fu G., Pan C., Wang S. (2018). Ultrahigh, Ultrafast, and Self-Powered Visible-Near-Infrared Optical Position-Sensitive Detector Based on a CVD-Prepared Vertically Standing Few-Layer MoS_2_/Si Heterojunction. Adv. Sci..

[181] Qiao S., Cong R., Liu J., Liang B., Fu G., Yu W., Ren K., Wang S., Pan C. (2018). A vertically layered MoS_2_/Si heterojunction for an ultrahigh and ultrafast photoresponse photodetector. J. Mater. Chem. C.

[182] Kim H.S., Kumar M.D., Patel M., Kim J., Cho B., Kim D.H. (2017). High-performing MoS_2_-embedded Si photodetector. Mater. Sci. Semicond. Process..

[183] Wu D., Lou Z., Wang Y., Yao Z., Xu T., Shi Z., Xu J., Tian Y., Li X., Tsang Y.H. (2018). Photovoltaic high-performance broadband photodetector based on MoS_2_/Si nanowire array heterojunction. Sol. Energy Mater. Sol. Cells.

[184] Lou Z., Zeng L., Wang Y., Wu D., Xu T., Shi Z., Tian Y., Li X., Tsang Y.H. (2017). High-performance MoS_2_/Si heterojunction broadband photodetectors from deep ultraviolet to near infrared. Opt. Lett..

[185] Dhyani V., Dwivedi P., Dhanekar S., Das S. (2017). High performance broadband photodetector based on MoS_2_/porous silicon heterojunction. Appl. Phys. Lett..

[186] Dhyani V., Das S. (2017). High-Speed Scalable Silicon-MoS_2_ p-n Heterojunction Photodetectors. Sci. Rep..

[187] Kim H.S., Kumar M.D., Kim J., Lim D. (2018). Vertical growth of MoS2 layers by sputtering method for efficient photoelectric application. Sens. Actuators A Phys..

[188] Guo J., Li S., Ke Y., Lei Z., Liu Y., Mao L., Gong T., Cheng T., Huang W., Zhang X. (2020). Broadband Photodetector based on vertically stage-liked MoS_2_/Si heterostructure with ultra-high sensitivity and fast response speed. Scr. Mater..

